# Exploring Strategies for a Digital Tool to Support Medication Adherence Among Adolescents and Young Adults Undergoing Hematopoietic Stem Cell Transplant and Their Care Partners: Qualitative Formative Study

**DOI:** 10.2196/82356

**Published:** 2026-02-17

**Authors:** Gavin Raab, Zoe Bowen, Skyla Shea, Guy Shani, Michelle Rozwadowski, Susan Allbritton Murphy, Inbal Billie Nahum-Shani, Ziping Xu, Alexandra Psihogios, Sung Won Choi

**Affiliations:** 1 Department of Pediatrics University of Michigan Michigan Medicine Ann Arbor, MI United States; 2 Strategy Area Stephen M. Ross School of Business University of Michigan Ann Arbor, MI United States; 3 Departments of Statistics and Computer Science Harvard University Cambridge, MA United States; 4 Institute for Social Research University of Michigan Ann Arbor, MI United States; 5 Department of Medical Social Sciences Northwestern University Feinberg School of Medicine Chicago, IL United States; 6 Rogel Cancer Center University of Michigan Michigan Medicine Ann Arbor, MI United States

**Keywords:** adherence, adolescent, family caregiver, family relations, hematopoietic stem cell transplant, medication, mHealth, mobile health, young adult

## Abstract

**Background:**

Allogeneic hematopoietic stem cell transplant (HCT) is a complex but essential treatment for malignant and nonmalignant conditions, requiring strict posttransplant adherence to immunosuppressant medications to prevent complications such as graft-versus-host disease. Adolescents and young adults undergoing HCT face unique challenges, including balancing growing independence with ongoing reliance on care partners, often parents. Medication adherence in this group is often suboptimal, and few interventions address adolescent and young adult–care partner dyads. To address this gap, we aim to develop a mobile health (mHealth) app that engages both the patients and care partner to improve adherence.

**Objective:**

As formative research for early-stage intervention development, this study aimed to (1) explore current HCT medication adherence strategies and challenges; (2) understand attitudes toward digital technology, including dyadic perspectives on app use to support adherence; and (3) assess adolescent and young adult–care partner relationships, including views on care partner involvement. This process was intended to inform the design of a relevant, user-centered mHealth app.

**Methods:**

Eligible participants included adolescents and young adult patients aged 12-39 years and primary care partners, such as parents, involved in medication management. Participants were recruited from a large academic medical center through direct outreach and electronic health records. Data collection involved 2 focus groups (6 dyads and 2 additional adolescents and young adults), 4 individual interviews (2 patients and 2 care partners), and 6 dyadic interviews. Semistructured sessions (in person or virtual) gathered feedback on medication adherence practices and app design preferences. All sessions were audio recorded with consent and professionally transcribed. Qualitative data were analyzed systematically: transcripts were deidentified, coded using both inductive and deductive strategies, and themes were refined through team consensus. Patterns were organized into major themes, and representative quotations were selected to illustrate findings. Data management was facilitated by NVivo (version 13; Lumivero) software.

**Results:**

We included 28 participants (15 adolescents and young adults and 13 care partners). The median age of adolescents and young adults was 18 (range 13-39) years and 53% (8/15) were female. Adolescents and young adults were 47% (7/15) White, 40% (6/15) Black, and 13% (2/15) mixed race. Care partners’ median age was 48 (range 36-72) years, with 92% (12/13) female and 77% (10/13) White. Three principal themes emerged: (1) existing reminders and organizational tools are often insufficient for consistent adherence; (2) adherence barriers are multifaceted, often involving autonomy vs care partner support; and (3) both adolescents and young adults and care partners showed strong interest in a dyadic digital health intervention to foster collaboration and support shared adherence goals.

**Conclusions:**

This formative study highlights the complex dynamics of medication adherence in adolescent and young adult–care partner dyads and supports the need for a dyadic mHealth app to enhance adherence, collaboration, and relationship quality.

## Introduction

Allogeneic hematopoietic stem cell transplant (HCT) is a potentially lifesaving treatment for malignant and nonmalignant conditions, such as aplastic anemia and sickle cell disease [1]. This procedure requires intensive radiotherapy and/or chemotherapy to achieve adequate immunosuppression, enabling successful engraftment of healthy donor stem cells [2]. Adolescents and young adults undergoing HCT face unique challenges, including disruptions in peer and family relationships, concerns about cancer care quality, and difficulties maintaining treatment-related health behaviors [3]. Adherence to immunosuppressant regimens (eg, tacrolimus and cyclosporine) is essential to prevent and treat potentially life-threatening posttransplant complications, such as acute graft-versus-host disease (GVHD) [4], with even minor deviations associated with poor clinical outcomes in transplant recipients [4]. Despite this, to our knowledge, no evidence-based interventions are available to support adolescents and young adults and their care partners, often parents, in medication adherence [5].

GVHD remains a leading cause of transplant-related morbidity and mortality among HCT recipients [6]. GVHD arises from a “cytokine storm” following host tissue injury, eventually resulting in target end-organ damage [7]. This complication can lead to cognitive decline, diminished health-related quality of life (HRQoL), and challenges reintegrating into society as a long-term survivor [8]. Thus, interventions aimed at improving adherence to prophylactic immunosuppressant medications could be especially beneficial for this population. This is especially relevant among adolescents and young adults, who show lower medication adherence than younger children across various conditions, including cancer [9], and who must navigate illness during a critical developmental stage [10].

In this population, we define medication adherence primarily as the success rate at which someone takes their prescribed medication. Of equal importance is the timing of the medication, 9 AM and 9 PM, for example, as the medication is most useful when taken at specified times (12 hours apart). Subsequent dimensions, such as routine formation, autonomy of medication taking, and collaboration with a care partner, also contribute to our definition. Medication adherence rates in the adolescent and young adult HCT population are suboptimal, estimated at 52%-72% [11]. Although understanding the specific risk factors and barriers to nonadherence is crucial, it remains significantly understudied in this group [11]. Existing research points to multiple reasons for missed doses, including medication refusal, resistance to care partner involvement, forgetfulness, side effects, and interference with usual activities such as socializing [11,12]. Adolescents and young adults have also expressed a desire for resources and tools, such as medication reminder systems, to support adherence [12].

Our prior research has shown that adolescents and young adults undergoing cancer treatment often rely on, or share, medication management responsibilities with their care partners [13,14]. Although adolescent and young adult years are characterized by increasing autonomy, many adolescents and young adults benefit from continued support due to the complexity of their treatments and associated side effects. It is also normative for adolescent and young adult-care partner dyads to experience relational strain during these years, as family roles and responsibilities are renegotiated to support the developmental strivings of adolescence (eg, peer affiliation and greater independence). While such challenges are often temporary, disruptions in family functioning can hinder medication adherence. A meta-analysis found that greater family conflict, lower cohesion, and poorer problem-solving were all associated with reduced adherence to medical regimens in children and adolescents with chronic health conditions [15].

Because HCT medication management typically requires active involvement from both the adolescent or young adult and care partner, the bidirectional relationship within the dyad is critical to consider [16]. The experiences and psychological well-being of both members are closely linked, and addressing them cohesively can improve medication adherence and related outcomes, such as HRQoL [17,18]. Interventions that engage care partners or focus on family functioning have been shown to improve medication adherence in pediatric and adolescent populations [19]. Dyadic models emphasize joint problem-solving and treating health care as a team effort [17], with interventions often incorporating behavioral strategies (eg, reminders and incentives), cognitive approaches (eg, shaping attitudes toward adherence), and collaborative problem-solving [19,20]. Thus, developing a digital intervention that targets the dyadic unit, rather than focusing solely on the patient or care partner, may yield a more effective approach [17].

This study is part of a multiphase research project to guide the early-stage development of a mobile health (mHealth) app delivering a dyadic just-in-time adaptive intervention (JITAI) for adolescent and young adult HCT recipients and their care partners. Traditionally, JITAIs provide timely, tailored support to individuals based on dynamic needs and contexts [21]; for example, offering medication reminders when an adolescent or young adult is away from home and at higher risk of forgetting. Mobile platforms are ideally suited for these interventions, enabling real-time personalization through continuous assessment of temporal contexts through sensors and brief surveys. Our proposed JITAI is uniquely designed to address the needs of both members of the adolescent and young adult–care partner dyad, supporting shared challenges and leveraging their interdependent experiences to promote medication adherence and well-being.

As formative research to inform app development, this study reports on findings from qualitative interviews and focus groups conducted with adolescent and young adult–care partner dyads. The semistructured sessions focused on three primary objectives: (1) exploring current HCT adherence and medication management strategies and challenges; (2) understanding attitudes toward digital technology, including dyadic perspectives on using an app together to support adherence; and (3) assessing adolescent and young adult–care partner relationships, including adolescents’ and young adults’ attitudes about care partner involvement in medication management. These sessions also incorporated a think-aloud approach [22] with hand-drawn mock-ups, enabling participants to provide feedback and co-design suggestions for the app’s features, layout, and design.

## Methods

### Study Design and Setting

**Figure 1:**
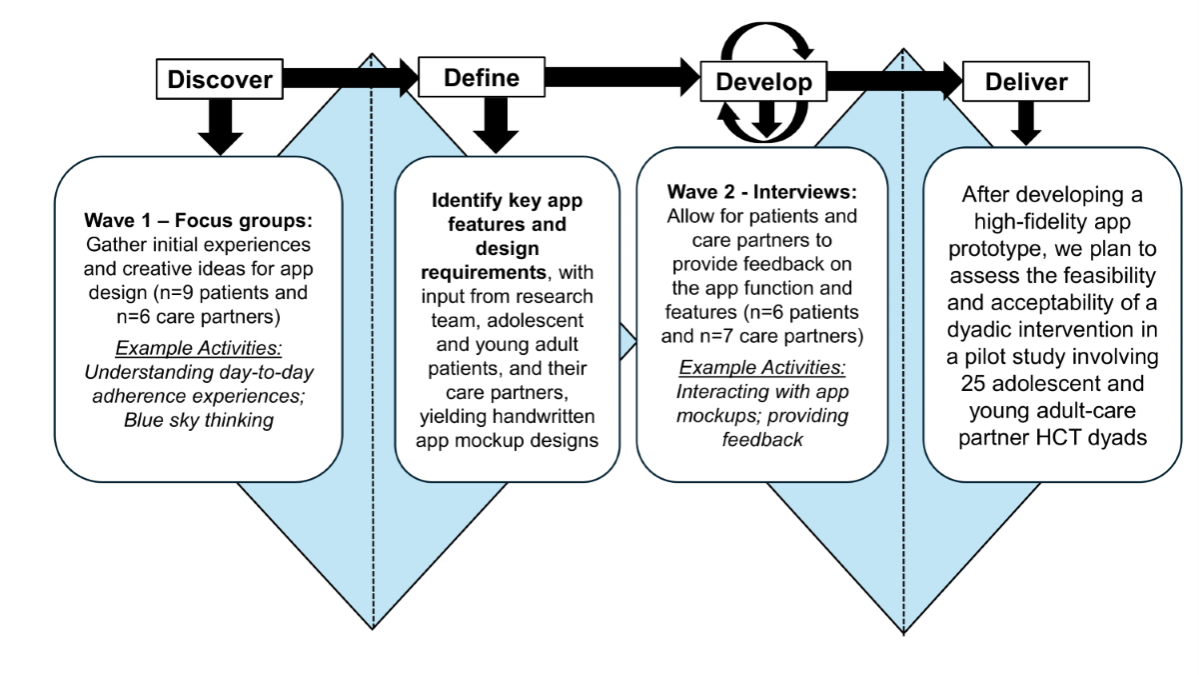
Double Diamond Framework. The “Discover” step is connected to Wave 1 focus group findings; the “Define” step is connected to identifying key app features and design requirements; the “Develop” step is connected to patient and care partner feedback that informs app development; and the “Deliver” step is connected to the planned future pilot study. HCT: hematopoietic stem cell transplant.

The study was conducted at a large, tertiary academic center in the Midwest (Michigan Medicine in Ann Arbor, Michigan, United States). Our methods were guided by the Double Diamond Framework (Figure 1), a widely used, human-centered design framework that incorporates both divergent (free-flowing and creative ideation, ie, the widening of the diamond) and convergent thinking (identifying concrete solutions, ie, the narrowing of the diamond) to co-create desirable and usable interventions with users [23,24].

### Recruitment and Enrollment

Eligible patients were aged 12-39 years, received care from a Michigan Medicine provider in the Pediatric Hematology/Oncology and Blood and Marrow Transplant and Cellular Therapy Unit, and were currently prescribed daily immunosuppressant medications, such as tacrolimus or cyclosporine. While the typical adolescent and young adult age range is 15-39 years, as defined by organizations such as the National Cancer Institute and the American Cancer Society, eligibility was extended to include patients as young as 12 years, as they commonly own smartphones and are of assenting age. Care partners were eligible if they were aged 18 years or older and identified by the patient as a primary care partner. All participants were required to speak English. Eligibility was determined through Michigan Medicine’s electronic health record, Epic (MiChart). Recruitment methods included flyers placed in clinic rooms, outreach in the clinics, as well as direct phone calls and emails. Informed consent (or assent for minors) approved by the University of Michigan Medical School Institutional Review Board was obtained from all participants using either traditional paper forms or electronic platforms. Recruitment continued until thematic saturation was reached, as indicated by the absence of new information or emerging themes [25].

### Focus Groups and Interviews

The study consisted of focus groups and semistructured qualitative interviews, conducted either in conference rooms at Michigan Medicine or through password-secured Zoom (Zoom Video Communications, Inc), involving 28 participants (15 adolescents and young adults and 13 care partners). Data collection included 2 focus groups, comprising 6 patient–care partner dyads and 2 additional adolescents and young adults, 4 individual interviews (2 patients and 2 care partners), and 6 dyadic interviews. Sessions were facilitated by trained research staff (GS and SWC) using standardized Moderator Scripts (Multimedia Appendix 1), which was initially developed by GS and then iteratively refined after each round of focus groups or interviews. Before the commencement of the interviews, GS and SWC explained to participants the motivations and primary goals for the interview sessions (Multimedia Appendix 1).

In Wave 1, focus groups and 2 individual interviews explored initial experiences and generated ideas; in Wave 2, a total of 6 patient–care partner dyadic interviews and 1 individual interview were conducted to gather usability feedback on app mock-ups (Figure 1). All sessions followed open-ended questions designed to elicit detailed discussion about medication adherence experiences, strategies, reasons for missed doses, patient–care partner interactions and collaboration, attitudes toward technology, and desired app features to support collaboration and relationship building.

A think-aloud procedure [22] using hand-drawn app mock-ups was used to collect in-depth feedback about app design, layout, and features. Focus groups lasted approximately 45 minutes, individual interviews approximately 25 minutes, and dyadic interviews approximately 60 minutes. Participants received US $20 compensation on completion. Sociodemographic data (eg, age, sex, disease name, and date of birth) were collected with permission through the electronic health record. All sessions were audio recorded and professionally transcribed verbatim by Babbletype Inc (Philadelphia) with permission; a second note-taker (GR and ZB) was present during the focus groups and interviews. All data were deidentified, and participants were assigned a unique code to maintain anonymity (deidentified transcripts are available upon request from the corresponding author).

### Thematic Analysis

Guided by the Double Diamond Framework [23,24], data were analyzed using a thematic approach [26], with ongoing integration between data collection and analysis. The codebook was developed by ZB and GR under the supervision of SWC. Initial coding was open-ended and conducted independently by both coders to identify broad patterns, using line-by-line or paragraph-level review of transcripts in NVivo 13 (QSR International). This stage focused on qualitative content analysis [27] and allowed for the generation of new codes as significant concepts and themes emerged. The team then compared and discussed their codes, collaboratively organizing them into an initial codebook (Multimedia Appendix 2). Notably, divergence in participant perspectives allowed refinement of the coding framework by expanding and clarifying code definitions. Convergence, in which similar patterns or themes emerged, helped prioritize and emphasize core themes in the analysis, allowing focus on commonly shared needs and concerns expressed by participants.

Analysis followed established phases of thematic analysis: familiarization with the data, generation of initial codes, searching for themes, reviewing themes, defining and naming themes, and producing the final report. As subsequent transcripts were analyzed, the codebook was iteratively refined, and new codes were added as warranted. Emerging patterns and subthemes were grouped into overarching themes, with short codes assigned to representative quotations. Each transcript was reviewed by at least 2 research team members (ZB, GR, or SS), and discrepancies in coding or theme interpretation were resolved by a third reviewer as needed. The final synthesis and interpretation of codes and themes were completed by ZB and GR and reviewed by the broader study team (SS, AP, MR, GS, and SWC). This iterative approach ensured that consistent, hierarchically organized themes were confirmed by later interviews and represented the key patterns across the dataset.

### Reflexivity

We are an interdisciplinary research team based at a tertiary academic medical institution in the Midwest, bringing diverse expertise grounded in biomedical sciences. Our research team is composed of a variety of racial and ethnic backgrounds, sexes, ages, and training levels. As noted in the Authors’ Contributions section, the research scientists hold PhD degrees in strategy (GS), behavioral and management sciences (IBNS), statistics (SM and ZX), and psychology (AP), with tenure-track appointments at academic institutions across the Midwest, South, and Northeast. The research assistants (GR, ZB, and SS) and lab manager (MR) have all been trained in qualitative research in the biomedical setting primarily by an MD physician-scientist with expertise in cancer and HCT research (SWC). Our team developed expertise in qualitative research through interdisciplinary programs and research projects. Our team’s collective experience shaped this research project, bringing together different and diverse perspectives and training. The moderator scripts were developed by GS, who has expertise in human factors research. Thus, the interpretation of the study findings is informed by our team’s clinical and scientific training, which shapes our perspective on medication adherence and digital health research. We acknowledge that our biomedical orientation influences how we frame, analyze, and contextualize the results.

### Ethical Considerations

The study was conducted in accordance with the Declaration of Helsinki (as revised in 2013). Ethics approval for the study was obtained from the University of Michigan Medical School Institutional Review Board (HUM00224785). Participants did not have any prior relationship with the research staff before consenting and completing the interview. All study participants provided informed consent by signing and returning an institutional review board–approved consent document. Each participant received US $20 compensation after completing the interview. Each participant was assigned a unique study ID to protect their identities, and all transcripts were deidentified prior to analysis. Data generated in this study were secured in a password-protected, HIPAA (Health Insurance Portability and Accountability Act)-compliant database. The authors take full accountability for the work, ensuring that the study was conducted with full integrity and that all questions or concerns related to the study were thoroughly investigated and addressed.

## Results

### Wave 1 Insights on Adherence Challenges and App Design Feedback

Table 1 summarizes the goals, major themes, participant quotations, and subsequent design decisions from Wave 1 of the study. It highlights current adherence challenges, attitudes toward technology, and patient–care partner collaboration, linking user insights to medication adherence strategies.

**Table 1:** Wave 1 goals, major themes, participant quotations, and Wave 2 feedback.

Theme	Wave 1: goals	Wave 1: major themes	Wave 1: quotes	Wave 2: design decisions and initial feedback
Theme 1 (correlation with medication adherence dimensions): strategies, such as pill boxes, phone alarms, and reminders, correlate with dose-taking and dose-timing dimensions of medication adherence.	Goal 1: current adherence challenges and strategies	Challenges: getting out of routine, sleeping in or staying out late, and living the life of a teenager (eg, spending time with friends). Strategies: pill boxes, phone alarms, or reminders.	“Reminders on my phone. I have an alarm clock as well” [MA-20 AYA] “I pair my medicine with a daily activity” [MA-06 AYA] “Life in general. A busy work schedule, other activities.” [MA-14 Care Partner] “Morning is a struggle.” [MA-17 Care Partner] “I’d usually be out socializing” [MA-02 AYA]	Patients self-reporting a dose being taken empowers autonomy and reduces direct care partner oversight. Adaptive daily reminders to play a game that implicitly reminds the patient to take their dose. Patients and care partners felt that these types of reminders would not be a burden.
Theme 2 (correlation with medication adherence dimensions): attitudes toward using technology, such as allowing patients to keep track of their medication on their own, correlate with medication-taking, routine, and habit formation, and autonomy surrounding medication adherence.	Goal 2: attitudes toward using technology	Technology would help adherence. The app should be visually appealing. It would make life easier. It allows patients to keep track of medication on their own.	“I think it’s better than having somebody…remind you…you still have that feeling of autonomy.” [MA-05 AYA] “I would feel like if the app is more eye-catching and attention catching, it would help.” [MA-06 AYA] “The value, to me, is that one, I can see it, but then she is also learning to be more independent with it, so when she is on her own, it’ll become a better habit that she’ll continue.” [MA-07 Care Partner]	A simple, user-friendly interface with bright colors. A calendar view available to both the patient and care partner provides some oversight while fostering adolescent and young adult independence.
Theme 3 (correlation with medication adherence dimensions): positive feelings from both patients and care partners toward collaborating in an app correlate with routine and habit formation and collaboration with a care partner.	Goal 3: attitudes toward patient and care partner collaboration in an app	Liking the idea of collaborating in an app. Believing it would be useful for the parent and patient to use the same app. Various levels of collaboration would work at different patient ages.	“I think that’s good, because that can, in some ways, push you to do better.” [MA-02 AYA] “I feel like that would help both of us get an understanding of what needs to happen.” [MA-06 AYA] “You don’t get too many times to sit there and collaborate on things too much.” [MA-10 AYA]	A collaborative word game that requires input from both patient and care partner. This feature leads to improved management of the dyadic unit, focusing on a common goal of completing the task. It takes dyadic communication focus away from medication and toward a collaborative pursuit.

### Demographics of Study Participants

The median age of the adolescents and young adult patients was 18 (range 13-39) years, with a balanced sex distribution of females (8/15, 53%) and males (7/15, 47%). Their racial distribution included 47% (7/15) White, 40% (6/15) Black, and 13% (2/15) mixed race. Among the care partners, the median age was 48 (range 36-72) years, with the majority being female (12/13, 92%). Racial distribution among care partners was 77% (10/13) White and 23% (3/13) Black. A detailed distribution of participants by focus groups and interviews is provided in Multimedia Appendices 3 and 4, respectively.

Table 2 summarizes demographic characteristics of the study participants, including gender, age, race, and ethnicity, for both patients and care partners.

**Table 2:** Patient and care partner demographic information.

Characteristic		Patients (n=15)	Care partners (n=13)	Total (N=28)
**Sex**				
	Male	7 (47%)	1 (8%)	8
	Female	8 (53%)	12 (92%)	20
Age (years), median (range)		21 (13-39)	48 (36-72)	33 (13-72)
**Race**				
	Black or African American	6 (40%)	3 (23%)	9
	White	7 (47%)	10 (77%)	17
	Mixed race	2 (13%)	0 (0%)	2
**Ethnicity**				
	Hispanic or Latino	0 (0%)	0 (0%)	0
	Non-Hispanic or Latino	15 (100%)	13 (100%)	28

### Focus Group and Interview Findings

#### Overview

**Figure 2:**
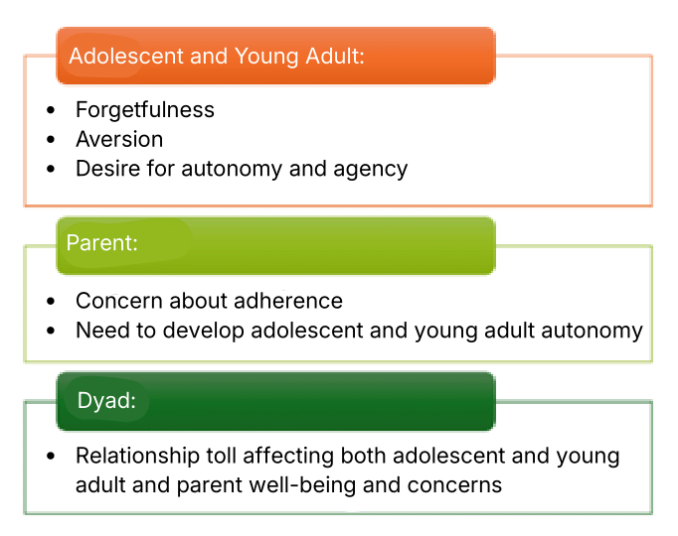
Key findings from focus groups and interviews.

The focus groups and interviews revealed key insights into the challenges, relationship dynamics, and strategies used by adolescents and young adults and their care partners in managing medication adherence, as well as their use of technology and preferences for an mHealth app. Major themes related to adherence barriers and challenges, as reported by patients, care partners, and dyads, are summarized below and illustrated in the schematic presented in Figure 2.

Adolescents and young adults perspective: patients commonly identified forgetfulness, medication side effects, and a sense of being constantly monitored by care partners (often parents) as barriers, leading to an increased desire for autonomy and agency.

Care partner (parent) perspective: care partners focused on ensuring medication adherence while also supporting the patient’s development of autonomy and self-management skills.

Dyadic perspective: both adolescents and young adults and care partners shared the goal of promoting adolescent and young adult autonomy and agency, highlighting the importance of balancing support with independence.

#### Medication Adherence Challenges

Adolescents and young adults consistently reported difficulties maintaining their medication regimen, with the most significant barrier being disruption of routines typical in this developmental stage. Common scenarios included staying out late with friends or sleeping in, which often resulted in missed or late doses.

For me, I’d usually be out socializing, so an event at a friend’s house or something, and then 9:00 rolls around and… I don’t have my [medication].

MA-03 AYA

Adolescents and young adults acknowledged that irregular schedules posed ongoing challenges to adherence, particularly for morning doses.

One big reason that I miss a dose is sleeping in. I like to sleep in. A lot of the time, I sleep in past 12:00 p.m., so I'll miss that morning dose, but I typically won't miss the evening dose.

MA-20 AYA

Their care partners also recognized that routines can be disrupted by staying up late and sleeping in.

I don’t know if it’s because he felt…It’s more or less just forgetting and being busy, the busier he is. If he is out with his friends or he is at work or something, it just slips his mind….We haven’t taken the tacrolimus yet. He’s done Xarelto like that though where he had the blood clots. He had to take that on a regimented basis. There were times when he just completely missed a dose. You’d be like, “Why?” He was just, “Oh, I forgot,” or, “I slept in,” or, “I wasn’t here and I didn’t have the pill with me.” That’s another big one is it’s time to take it and not having it with them.

MA-02 Care Partner

#### Existing Strategies for Medication Adherence

Patients and care partners described several strategies used to support adherence, including setting phone alarms, using reminder and calendar apps, and organizing medications in pill boxes. These tools helped automate reminders and allowed care partners to discreetly monitor adherence, reducing the need to directly prompt the patient.

Timers are really good to help remembering to take your meds…I have an iPhone, but the Reminders app is okay. Then, what I will also do, sometimes, is input events in my Calendar app on my phone. It will pop up, and you can set it to remind you every day or every week or whatever the case may be. Those are three helpful things electronically.

MA-05 AYA

I do like the pillbox though because then I can always double check, you did, in fact, take it, without having to bug her constantly.

MA-06 Care Partner

Despite these efforts, both adolescents and young adults and care partners shared that busy schedules, forgetfulness, and the desire to avoid being constantly reminded still contributed to suboptimal adherence.

No, they’re just like, ‘No big deal.’ We’re the ones hovering over the bedside, watching them suffer, and they’re knocked out. They don’t remember half of it.

MA-04 Care Partner

I’ll say if I’m having a really busy day and I don’t keep track of everything that I’m doing or everything I need to do, I forget that I have to take my medicine. I’m like, ‘Oh, I forgot to take my medicine today’.

MA-06 AYA

### Views on Technology and Tools to Promote Medication Adherence

#### Attitudes Toward Using an App to Assist With Adherence

Care partners expressed enthusiasm for the potential of an app to support medication adherence.

Oh my God, you guys can help so many, so many patients.

MA-12 Care Partner

Care partners highlighted the value of verifying dose completion without direct verbal reminders.

Just as a point that we can make sure they’ve taken it without having to constantly call them or text them or bug them about it, is where I see the value in it more.

MA-07 Care Partner

Adolescents and young adults shared this interest, particularly appreciating features that could foster a greater sense of autonomy.

I think it’s better than having somebody, like family or friend, remind you, only because, if it’s a reminder that you set up yourself, you still have that feeling of autonomy. You did yourself, it’s you reminding yourself, instead of somebody else.

MA-05 AYA

“It’d probably be better if it was an app or an inanimate object yelling at me, rather than my own mom. It’d be easier to deal with.

MA-02 AYA

#### Potential Concerns About App Features

Adolescents and young adults and care partners agreed that complex or time-consuming apps would be burdensome. Preferred features included a shared calendar for medication tracking, notification reminders, and optional shared activities, such as a simple and engaging game that could be completed in under 5-minutes. An interviewer asked adolescent and young adult participants and a care partner about the ideal length of a game within the app

Interviewer: How long do you guys think would be a good amount of time to have the game take?

MA-06 AYA: No more than five minutes.

MA-18 Care Partner: I know every person’s situation is different, but I work full-time. My husband works full-time, so adding this on top of all of the other responsibilities. Maybe it's an optional game within the app.

#### Overall Suggestions for App

Care partners expressed developing routines and strategies early in the transplant process.

[My daughter] has been on it since she was five, so it was easier when she was younger, because I felt more responsible for it, but now that she’s older, and I’m trying to get her gear, it has been a little bit difficult.

MA-06 Care Partner

Care partners valued the ability to send reminders through the app, reducing the need for direct prompting and supporting the adolescents and young adults’ autonomy.

If we can go in the app and have the app send them a notification. Like, ‘Check and see if she took her meds today.’ I think that would be something.

MA-06 Care Partner

Both adolescents and young adults and care partners responded positively to a built-in reward system to help motivate and help support the patient’s continued, consistent medication adherence.

They get a streak or they get to a certain point and they get a little gift or they can send a postcard to their friends or whatever.”

MA-02 Care Partner

I would say that’s nice because, yes, we’ve taken it all our lives, but I feel like being rewarded for something makes you want to do something even more.

MA-06 AYA

The app could also facilitate community-building among adolescents and young adults and care partners with similar medical conditions, providing an additional source of support and relatability that families may not have previously experienced. An interviewer asked an adolescent and young adult participant about the possibility of connecting with others who have a similar disease.

Interviewer: Then maybe you could connect with other people who have a similar disease to you or something like that?

MA-06 AYA: Yes.

Additionally, participants suggested including a feature that allows adolescents and young adults to record reasons for missed doses (eg, an “other” or “why” box), enabling them to note why a dose was not taken. This could help adolescents and young adults identify patterns, such as specific activities or times of day when doses are missed, ultimately supporting improved adherence.

I feel like maybe there should be a fourth, like a “other” in there, just in case for some weird reason those three don't actually sum up what it is and you can actually type out what your “other” is.

MA-18 AYA

### Patient–Care Partner Dyadic Relationship Quality

Adolescents and young adults frequently described tense or frustrating interactions with care partners, often centered on medication reminders. Care partners similarly expressed reluctance to repeatedly prompt adolescents and young adults but felt their involvement was vital for successful medication management during the transplant process. The desire for increased autonomy and agency was a recurring theme for adolescents and young adults.

...sometimes when your parents are on you a lot, it can be annoying. It’s like when you’re young and you go to do the dishes. You’re on the way to the dishes, but while you’re on the way there, your parent says, “Go do the dishes,” and you don’t want to do the dishes.

MA-05 AYA

Care partners expressed similar feelings, noting a reluctance to repeatedly remind adolescents and young adults about medication adherence. Nonetheless, they viewed their support, including providing reminders, as essential for successfully navigating the transplant process.

It’s your medicine. I’m just here giving you a little helping and a little nudging, little kick butting. If there’s anything you think will work better, so we don’t have to go through this dance, you just throw it out there and let’s talk about it.

MA-04 Care Partner

### Patient’s Desire for Autonomy and Agency

Adolescents and young adults expressed a strong desire for autonomy and independence in managing their lives and reported frustration with frequent reminders from care partners about medication adherence.

She wanted to feel like she was in control of her life, not that the cancer took control of her life, so many times even if she took a dose she didn't want us to know that she took a dose.

MA-12 Care Partner

Sometimes my mom will get on me about taking it. That makes me actually want to take it less, because I find it annoying, but that’s not always the case. Sometimes I have legitimately forgotten, while other times it’s usually just a matter of being a kid and having a parent tell me what to do.

MA-02 AYA

### Care Partner Perspectives on Facilitating Medication Adherence

Care partners shared a range of emotions related to supporting medication adherence, from maintaining a positive outlook to experiencing fatigue, worry, and anxiety.

Her health, her staying alive, her fighting through this, her getting through the whole process, and she's my baby. I love her. I don't want anything bad to be happening to her.

MA-09 Care Partner

How I felt? Very worried, very anxious. (Laughs.) Yes. I actually personally had to go to my primary doctor and I asked for help and I had to take some Lexapro.

MA-12 Care Partner

### Perspectives About Using an App Focused on the Dyadic Relationship

Both adolescents and young adults and care partners responded positively to the idea of an app designed for use by both members of the dyad. They saw potential for the app to support adolescents and young adults’ autonomy as well as connection within the relationship through shared or collaborative management of the patient’s health.

The value, to me, is that one, I can see it, but then she is also learning to be more independent with it, so when she is on her own, it’ll become a better habit that she’ll continue.

MA-07 Care Partner

I feel like that would help both of us get an understanding of what needs to happen.

MA-06 AYA

### App Mock-Up Design Interviews

Findings from the focus groups and interviews informed the design of low-fidelity, paper-based mock-ups of the app intended for dyadic use (Multimedia Appendix 5). Participants expressed interest in a shared calendar view, in which adolescents and young adults could log their medication intake and care partners could view, but not edit, these entries. This could facilitate information flow between adolescents and young adults and care partners while maintaining adolescents and young adults’ autonomy. This approach centers medication management with adolescents and young adults, allowing them to update their logs while providing care partners with oversight through viewing access only (ie, care partners cannot enter themselves; rather, medications are managed by adolescents and young adults; Table 3).

**Table 3:** Medication log feedback.

Interviewer-asked question	Participant quotes
“How do you feel about using one of those apps with a care [giver] or parent?”	“I think that would be really helpful because as much as I’m in control of mostly everything I take, it would feel even better if I could just log it myself without making it more of a pressure for my parents to log.’ [MA-17 AYA]
“...and then you would be able to see the exact time she took it because she’ll click that she took it at that time and it’ll log the time that she took the medication.”	“Oh my god, you guys can help so many, so many patients ... .Oh, this is really good, ‘Medication Log’ I think it looks good, yes.” [MA-12 Care Partner]
“Describe what you see from top to bottom. What pops up to you as something that you notice?”	“I think the medication log is really cool, just to keep track of what medication you take on whatever day, or how much you have to take, or just even the messages.” [MA-2 Care Partner]

Adolescents and young adults and care partners agreed that working together or collaborating on a shared game within the app could enhance their relationship quality and provide a positive distraction from illness management and related stressors. Participants emphasized that the game should require minimal time commitment, as adolescents and young adults often experience attention and cognitive fatigue, especially over extended periods (Table 4).

**Table 4:** General game feedback.

Interviewer-asked question	Participant quotes
“What did you like about it (the game)?”	“I like the idea. The game draws in the parent and the child, tracking. You go into it to play the game, but you also are tracking your medicine.” [MA-14 Care Partner] “I think it’s a mind distraction too. Instead of me, ‘Come on, [AYA], take the meds, take the meds,’ it’s, ‘Okay, as she’s taking the meds, we’re playing a game.’ We’re doing something more interactive.” [MA-17 Care Partner] “Yes, mm-mmm (agreement).” [MA-17 AYA]

The design goals included creating a low-effort, collaborative game for adolescents and young adults and care partners; for example, a word-solving activity in which each member of the dyad (eg, adolescent or young adult and care partner) is assigned specific words within the phrase. Importantly, this collaborative opportunity would take place outside of medication management. The game is intended to be noncompetitive, fostering mutual benefit and possibly strengthening the dyadic relationship by engaging in a shared activity they are doing together (Table 5).

**Table 5:** Collaborative game feedback.

Interviewer-asked question	Participant quotes
“You have your own word that you can solve…behind the scenes you can meet up together and solve both words together.”	“I like it. I just was wondering. It’s intriguing. I think it’s fun…Even if they’re too young to have the app themselves, they can do it on their parent’s phone and they can input, by just touching the screen and the parents can talk over it and say, ‘Okay, we’re going to input your dose. I’ll help you input it.’ I like it!” [MA-15 Care Partner] “If they’re just constantly being reminded to take pills, that’s (the game) maybe another way that can make you…maybe just a little bit more fun instead of just being like, ‘Oh, it’s time to take this.’” [MA-2 AYA] “You don’t feel like you’re nagging as much because I feel like I nag, but that’s my job, so that’s what I do (laughs).” [MA-2 Care Partner]

## Discussion

### Principal Findings

This qualitative study showed that adolescents and young adults and their care partners face significant challenges in maintaining adherence to their immunosuppressant regimens, despite the essential role of these medications in preventing serious posttransplant complications. Although adolescents and young adults strongly desired autonomy and agency in managing their medications, our findings indicate that when left to self-manage, they frequently missed doses due to disrupted routines, oversleeping, forgetfulness, side effects, and competing priorities typical of this developmental stage, such social activities. These findings are consistent with recent studies reporting lifestyle (ie, adjusting to new social and family dynamics) and sleep as common post-HCT challenges [28-30]. In adapting to these challenges, both adolescents and young adults and care partners relied on strategies like pill boxes, phone alarms, and reminder systems. However, these tools often failed to fully overcome barriers such as busy schedules and shifting routines (eg, clinic appointments, transfusions, and other infusions). Eventually, adoption of strategies could help build habit formation, which was similarly discussed in another qualitative study among patients living with acute GVHD: “transitioning from an external obligation to a habit” [31].

When specifically asked, participants viewed technology positively, expressing enthusiasm for a simple, user-friendly app that both adolescents and young adults and care partners could use to support medication tracking, offer visual confirmation of adherence, and provide motivational features, such as rewards or messages. These findings align with recent digitally-delivered interventions reported in similar, though not exclusively HCT, patient populations with cancer [32-35]. Adolescents and young adults preferred app-based reminders over direct prompts from care partners, suggesting that technology may serve as a neutral facilitator of adherence, supporting their need for autonomy and agency, a central developmental goal for this age group [29,36]. Lastly, using low-fidelity mock-up designs, both adolescents and young adults and care partners valued features that balanced oversight with independence, such as a shared medication calendar, optional reminders, and collaborative, low-effort activities (eg, brief games) to support adherence and strengthen their relationship. These insights have collectively informed the initial co-design of a dyadic mHealth app with shared features and activities that both adolescents and young adults and care partners could use to promote medication adherence. This intervention is intended to mainly impact dose-taking and dose-timing, with additional improvements in patient autonomy, routine and habit formation, and collaboration with care partners.

### Comparisons and Contrasts With Previous Research

Our findings support previous research indicating that medication nonadherence remains a significant concern among HCT recipients. A literature review of 5 studies on medication adherence in the HCT population reported rates of adherence ranging widely from 33% to 94% across pediatric and adult populations, with a trend toward declining adherence over time [30]. More recent studies further highlight this issue: a prospective survey of 200 adult allogeneic HCT recipients found that nearly 38% reported medication nonadherence to oral immunosuppressants, and 51% had nonadherence to nonimmunosuppressants closely linked to complex regimens, frequent medication adjustments, younger age, polypharmacy, fatigue, and psychological distress [37,38]. Similarly, a cross-sectional study of 203 adult and 39 pediatric allogeneic HCT recipients reported a nonadherence rate of 75% to cyclosporine [39]. Among long-term survivors, another report identified 69% nonadherence to immunosuppressants, highlighting the persistence of adherence challenges even among those without active GVHD or even with mild chronic GVHD (62%), as well as those with more severe presentations (80%) [40]. Taken together with our qualitative formative research, these studies provide strong rationale for addressing critical gaps through design strategies and behavioral interventions to support medication adherence.

Indeed, our own data reinforce concerns about nonadherence, identifying barriers such as oversleeping, busy routines, side effects, and, particularly relevant for adolescents and young adults, ongoing negotiation between care partner oversight and adolescents and young adults’ desire for autonomy. These challenges support established literature [41]. Care partners play an essential but often stressful role, trying to balance support with allowing for adolescents and young adults’ independence. A recent mHealth app (BMT4me) was developed with this in mind to promote adherence to immunosuppressant medication and track symptoms in children following HCT [35,42]. In their recent pilot study, the authors reported that care partners found the app helpful in organizing medications, setting reminders, tracking symptoms, and emphasized the value of digital support during an overwhelming phase of early post-HCT care (ie, the first 100 days). However, they noted the challenge of maintaining sustained app engagement over time, with some users never logging in after the initial onboarding [35].

### Unique Contributions of This Qualitative Study

Studies by Amonoo et al [38] and Kochashvili et al [35] highlight the crucial role of care partners in posttransplant care, especially in medication management during the early acute postdischarge phase when patients are often fatigued, cognitively taxed, and psychologically distressed. Their findings show that care partner involvement is vital for establishing and sustaining medication routines, as supported by representative qualitative quotes from both studies. While the recent BMT4me app study demonstrates the impact of a digital app specifically for care partners of patients aged 2-18 years, showing user satisfaction, usefulness of reminders, and establishing new habits posttransplant, our qualitative research extends this work in several unique ways. Our findings emphasize the dyadic relationship [16] and support the design of an intervention that targets the dyadic unit, engaging both adolescents and young adults and care partners as active participants [43]. Accordingly, we included both members of the dyad in focus groups and interviews, including input from both in the co-design of using low-fidelity app mock-ups. Our participants clearly articulated a desire for features that promoted both autonomy and relational connection. Our emphasis on strengthening the dyad, not only in practical management (eg, adolescents and young adults self-reporting), but also through relationship-building features (eg, shared calendar and games), is an innovation. Our formative research specifically identified the value of simple, brief, noncompetitive, collaborative games as both a motivator for app use and a means for shifting care partner communication toward positive, goal-directed interactions, moving away from framing medication as a source of “nagging” or conflict. This concept of gamification aligns with recent findings of Tran et al [44] in their scoping review, which synthesized support on the role of incentive- and game-based features in mHealth adherence apps. Their work indicated the importance of early and meaningful involvements of patients in app design phases. This study directly responds to this recommendation by engaging both adolescents and young adults and care partners in the formative research and design process (ie, co-design) to ensure that gamification features and engagement are meaningful, developmentally appropriate, and tailored to the unique needs and daily lives of posttransplant dyads. For example, our participants provided input on features that could motivate continued app interaction and emphasized the point of brief games (ie, 5-minutes or less), as highlighted in the review by Tran et al [44].

### Proposed Dyadic Just-In-Time Adaptive Intervention Components

Based on our formative qualitative interviews and established literature on JITAIs [21], we anticipate that our dyadic JITAI will incorporate the following elements. Decision points are times when the intervention delivery can be adapted based on the participant’s current state or context. For our dyadic intervention, decision points could be based on scheduled dose time (ie, morning/evening dose windows selected by the adolescents and young adults) or missed self-report detection (ie, failure to log a dose within a set time frame).

Potential tailoring variables are characteristics or real-time contextual features used to personalize the intervention. These may include previous medication adherence trends or patterns, such as logged dose status of “taken,” “missed,” or “paused” in the previous window, or average self-report missing rate in the past week; self-reported barriers, including “forgot,” “busy,” “out with friends,” “sleeping in,” “side effects,” or typed open-text reasons in an “other” box; dyad mood, physical illness, and relationship quality (ie, through in-app daily assessment or diary); or app engagement (ie, time and frequency of app use).

Potential intervention options include positively framed messages near scheduled medication dose times (ie, in lieu of actual medication reminders), game-focused reminders that engage the dyads in interacting with one another around scheduled medication times, or context-specific messaging. For example, when certain barriers are reported (eg, “forgot” or “busy”), motivational messages or tips (eg, “pair meds with your evening routine!”) could be sent. Additional options include dyadic support prompts; if low mood is detected, additional prompts could be sent that encourage or lift mood; rewards for consecutive adherence, delivered as shared app features (eg, unlock new game levels and send congratulatory postcards); or care partner engagement or information prompts. The care partner will be able to view the calendar (but not edit) to see whether repeated adherence lapses occur, with encouragement to use the collaborative game feature.

Thus, adaptation may occur based on time, if doses are consistently missed at a certain time (eg, morning), intervening with adjusted reminders or routine-change suggestions; context, if social activity, tiredness, or side effect reporting is high in a given period, adapting messaging or interventions accordingly; adherence behavior, whereby the app could increase support for dyads with declining adherence (eg, motivational messaging); dyadic interaction, whereby low engagement with the collaborative app features triggers prompts to remind dyad of benefits or to schedule shared activities. Thus, the initial intervention may rely on scheduled reminders, but as the app learns the individual’s barriers and the dyad’s engagement patterns with the game, it will provide contextually relevant supports, such as encouraging collaborative game play after missed doses and offering motivational, agency-preserving messages for adolescents and young adults when repeated “nagging” is detected as a source of friction. The proposed variables are founded in the JITAI principles described by Nahum-Shani et al [21] and the emergent findings of this formative qualitative study. They will be iteratively refined in a future pilot as more data are collected and may be further improved during a study on the fly through an optimization algorithm.

### Study Limitations

This study had several limitations. The sample size was relatively small and was drawn from a single academic institution, which may limit the generalizability of our findings to a broader and more diverse population of adolescents and young adults undergoing HCT. Participation was limited to English-speaking individuals, excluding non–English speakers who may face distinct adherence barriers and whose experiences are not reflected in this analysis. In addition, participants were compensated and tended to have favorable attitudes toward research, the health care system, and the health care being provided, which may have introduced selection or response bias. Our sample included adolescents and young adults with both cancer and noncancer hematologic conditions at various stages in their treatment trajectory, rather than focusing specifically on those within the acute period (first 100 days) posttransplant. As a result, our findings may not fully capture the unique challenges and experiences faced by adolescents and young adults during the most critical early phase following transplant. Future research should aim to include larger, more diverse, and geographically varied cohorts, as well as specifically study participants during the acute posttransplant period to enhance the relevance and applicability of findings. Developmental variability may have impacted the interpretation of our findings and potential applicability to a broader population.

### Conclusions

In summary, our findings highlight the need for innovative, dyadic interventions that actively engage both adolescents and young adults and their care partners as a dyadic unit in efforts to improve medication adherence. Despite decades of research documenting adherence challenges in the HCT population, existing interventions have often been limited in scope and struggled with low engagement, leaving adolescents and young adults especially underserved during this developmental stage. In this study, both adolescents and young adults and care partners showed strong interest and willingness to explore new approaches, expressing particular enthusiasm for digital health technologies to support medication management. By fostering autonomy in adolescents and young adults and strengthening collaboration within the dyad, a well-designed mHealth app has the potential not only to enhance medication adherence but also to improve HRQoL, well-being, communication, and overall outcomes for both adolescents and young adults and care partners. The formative insights presented herein provide a solid foundation for our upcoming pilot study using an mHealth app, which will test JITAIs delivering individualized and dyadic messages, as well as interactive, relationship-building activities such as brief games. Our goal is to promote sustained medication adherence and support dyadic relationship quality by actively involving both the patient and care partner in the intervention.

## Appendix

Multimedia Appendix 1 Moderator scripts.

Multimedia Appendix 2 Medication adherence focus group and interview codebook.

Multimedia Appendix 3 Patient and care partner demographic information–focus groups.

Multimedia Appendix 4 Patient and care partner demographic information–individual and dyadic interviews.

Multimedia Appendix 5 Low-fidelity, paper-based app mock-ups.

## Abbreviations

GVHD: graft-versus-host disease
HCT: hematopoietic stem cell transplant
HIPAA: Health Insurance Portability and Accountability Act
HRQoL: health-related quality of life
JITAI: just-in-time adaptive intervention
mHealth: mobile health

## Data Availability

The study protocol and data generated or analyzed (ie, anonymized interview transcripts) during this study are available from the corresponding author on reasonable request. The codebook is available in Multimedia Appendix 2.
