# Supplementary material for: Exploring Strategies for a Digital Tool to Support Medication Adherence Among Adolescents and Young Adults Undergoing Hematopoietic Stem Cell Transplant and Their Care Partners: Qualitative Formative Study
Source: JMIR Form Res. 2026 Feb 17;10:e82356. doi: 10.2196/82356 (PMC12957942; doi:10.2196/82356)
Supplement: Multimedia Appendix 1 [file formative_v10i1e82356_app1.docx]

**Multimedia Appendix I: Moderator Scripts**

# ADAPTS-HCT Focus group session 1 protocol [AYA version]

## Introduction

We are developing a new application to help adolescents and young adults (“AYA”) with taking Tacrolimus (or cyclosporine) during the early phase of transplant. We are focusing primarily on the first 100 days of transplant. The application is designed to be used by both the AYA and the parent/caregiver by providing prompts to help the AYA and their parent/caregiver with taking Tacrolimus (or cyclosporine) consistently and receive additional support when needed.

You can help with the development of this application by sharing your experiences with taking Tacrolimus (or cyclosporine). The purpose of the focus group is to gather feedback about how you remember to take your doses of Tacrolimus (or cyclosporine) and how your parent/caregiver supports you in doing so.

We will audio-record the focus group today. This will allow us to go back and listen carefully and make sure that we correctly capture what you said. We will also take notes, but these notes will not be identified by your name or other identifiers. The electronic files are also confidential and will not be shared with people outside of our research team. Some of the topics we talk about might be sensitive, you can skip any question if you feel uncomfortable answering. You can choose not to continue participation at any time during the focus group.

## Goal 1: Current adherence and medication management strategies and challenges [10-15 minutes]

1. Adhering consistently to taking Tacrolimus (or cyclosporine) can be a struggle for AYA.
2. What are examples of reasons for you missing a dose?
3. Is there a time of day during which you are more likely to miss a dose?
4. Can you describe a day during which you were more likely to miss a dose of (For example, days of the week; Events that were planned for the day; The way you felt during the day; Slept in too late).
5. Do interactions with parents/caregivers affect the likelihood of missing a dose? How so?
6. What do you do if you miss a dose?
   1. Who do you tell?
   2. How do you feel about missing a dose?
7. Are there things about taking Tacrolimus (or cyclosporine) that you would want to know more about?
8. What motivates you to keep taking your doses of Tacrolimus (or cyclosporine)?
9. Are there other health and mental health topics other than Tacrolimus (or cyclosporine) that are important to you?
10. **What strategies do you use to help remember to take your dose of Tacrolimus (or cyclosporine)?**
11. Is there someone that helps you remember to take your dose?
12. How do you feel about having someone help you remember to take your dose?
13. Do you prefer not to receive help or not?

## Goal 2: Attitudes toward using technology to help taking medication and health management [10-15 minutes]

1. Do you use any technology to help remember to take your dose? Do you set up alarms? Do you use an application on your phone? Do you keep a journal/diary to keep track?
2. Using apps and technology for health is becoming more common:
3. Do you use any apps to help with managing your health? For example, Fitbit, Apple Health, Samsung Health, or other applications?
   1. If so:
      1. Which do you use?
      2. How does it help you manage your health?
      3. Can you share an example of how it helps you manage your health?
   2. If not:
      1. Have you tried such an app in the past? If so, why did you stop?
      2. Is there a reason you do not use an app to help you manage your health?
4. How do you feel about using an app to help you remember to take your dose, and keep track of the doses that you took?
5. How do you feel about your caregiver/parent also using the application to help you remember to take your dose?
6. Sometimes there is a need to skip a dose of Tacrolimus (or cyclosporine):
7. How do you know when to skip a dose?
8. How do you keep track and remember when to take a dose, and when to skip it?
9. How do you feel about needing to skip a dose?
10. Is it difficult to remember to take a dose once you are no longer skipping a dose?
11. How do you communicate skipping a dose with your parent/caregiver?
12. There is new technology that helps keep a record of when you take a dose – for example a special pill bottle with a cap that records anytime you twist it open.
13. Would you want to use such a pill bottle to actively keep track of when you took your dose?
    1. If so, why?
    2. If not, why?

## Goal 3: Attitudes about your parents helping you with your medication and health management [10-15 minutes]

1. Would you mind if you and your parent both used an app that focused on playing a game together using an app that was geared toward you taking your medications?
   1. If so:
      1. Would you enjoy trying to solve a puzzle together?
      2. Would you feel burdened about getting prompts or reminders through the app about the game? Or about taking your medication?
      3. Do you mind getting text messages from your parents about medications? Can you describe how your parents currently remind you? What is the system you guys currently use?
      4. How many pings a day from an app would be too much?
      5. Would you mind completing a survey each day about your experience with the app? Or would you prefer weekly?

## Goal 4: Patient stories and “Blue Sky Thinking” activity [~15 minutes]

We will now read you two patient stories about taking a dose of Tacrolimus (or cyclosporine). We ask that each of you think of one creative idea for helping the person in the story to take their dose of Tacrolimus (or cyclosporine). After each of you shares your idea, we will go around again and give you the option to either “keep” the idea you shared, or to “steal” one of the other ideas that you like better. This will help us learn about different ways to help and understand if some ideas appear better than others.

1. **Story 1:** Jordan is a sixteen-year-old. He remembers to take his Tacrolimus (or cyclosporine) on weekdays, but usually forgets to take his doses during the weekend. During the weekends, Jordan is usually online till late because his friends can stay up and play with him online. After a long night of gaming, Jordan is tired and ends up waking up later in the day.
   *What is a creative idea you have for Jordan to help him with taking his Tacrolimus (or cyclosporine)?*
   *(after everyone shares) Would you like to keep your idea or steal one of the other ideas?*
2. **Story 2:** Sam is a seventeen-year-old. She is a senior in high school and feels lonely because she has been unable to participate in clubs she loves in school. She feels as if her mother is always around and asking her about taking her dose. When she takes Tacrolimus (or cyclosporine), it makes her feel ill and if her mother doesn’t remind her, Sam will sometimes skip a dose on purpose to forget about being sick and avoid feeling ill.
   *What is a creative idea you have for Sam to help her with taking her Tacrolimus (or cyclosporine)?
   (after everyone shares) Would you like to keep your idea or steal one of the other ideas?*

## Conclusion [~5 minutes]

Thank you and opportunity to share some final comments and ideas.

# ADAPTS-HCT Focus group session 1 protocol [Parent version]

## Introduction

We are developing a new application to help adolescents and young adults (“AYA”) with taking Tacrolimus (or cyclosporine) during the early phase of transplant. We are focusing primarily on the first 100 days of transplant. The application is designed to be used by both the AYA and the parent/caregiver by providing prompts to help the AYA and their parent/caregiver with taking Tacrolimus (or cyclosporine) consistently and receive additional support when needed.

You can help with the development of this application by sharing your experiences with supporting your AYA with taking Tacrolimus (or cyclosporine). The purpose of the focus group is to gather feedback about how you interact and support your AYA with remembering to take their doses of Tacrolimus (or cyclosporine).

We will audio-record the focus group today. This will allow us to go back and listen carefully and make sure that we correctly capture what you said. We will also take notes, but these notes will not be identified by your name or other identifiers. The electronic files are also confidential and will not be shared with people outside of our research team. Some of the topics we talk about might be sensitive, you can skip any question if you feel uncomfortable answering. You can choose not to continue participation at any time during the focus group.

## Goal 1: Current adherence and medication management strategies and challenges [10-15 minutes]

1. Adhering consistently to taking Tacrolimus (or cyclosporine) can be a struggle for AYA.
2. What are examples of reasons for your AYA missing a dose?
3. Is there a time of day during which your AYA is more likely to miss a dose?
4. Can you describe a day during which your AYA was more likely to miss a dose (For example, days of the week; Events that were planned for the day; The way the AYA felt during the day; Slept in too late).
5. Do interactions with you (parents/caregiver) affect the likelihood of your AYA missing a dose? How so?
6. What do you do if your AYA misses a dose?
   1. Who do you tell?
   2. How do you feel about your AYA missing a dose?
7. Are there things about taking Tacrolimus (or cyclosporine) that you would want to know more about?
8. What motivates you to keep supporting your AYA in taking their doses of Tacrolimus (or cyclosporine)?
9. Are there other health and mental health topics other than Tacrolimus (or cyclosporine) that are important to you?
10. **What strategies do you use to help your AYA remember to take their dose of Tacrolimus (or cyclosporine)?**
11. Do you play an important role in the AYA remembering to take their doses?
12. How do you feel about having to help the AYA remember to take their dose?
13. Do you prefer not to provide help or not?

## Goal 2: Attitudes toward using technology to help taking medication and health management [10-15 minutes]

1. Do you use any technology to help the AYA remember to take their dose? Do you set up alarms? Do you use an application on your phone? Do you keep a journal/diary to keep track?
2. Using apps and technology for health is becoming more common:
3. Do you use any apps to help with managing your own health? For example, Fitbit, Apple Health, Samsung Health, or other applications?
   1. If so:
      1. Which do you use?
      2. How does it help you manage your health?
      3. Can you share an example of how it helps you manage your health?
   2. If not:
      1. Have you tried such an app in the past? If so, why did you stop?
      2. Is there a reason you do not use an app to help you manage your health?
4. How do you feel about using an app to help the AYA remember to take their dose and keep track of the doses that they took?
5. How do you feel about you (as the caregiver/parent) also using the same application to help the AYA remember to take their dose?
6. Sometimes there is a need to skip a dose of Tacrolimus (or cyclosporine):
7. How do you know when your AYA needs to skip a dose?
8. How do you keep track and remember when the AYA needs to take a dose, and when to skip it?
9. How do you feel about your AYA needing to skip a dose?
10. Is it difficult to keep track of when your AYA needs to take a dose once they are no longer skipping a dose?
11. How does your AYA communicate skipping a dose with you?
12. There is new technology that helps keep a record of when a dose is taken – for example a special pill bottle with a cap that records anytime it is twisted open.
13. Would you want to use such a pill bottle to actively keep track of when your AYA took their dose?
    1. If so, why?
    2. If not, why?

## Goal 3: Attitudes about your parents helping you with your medication and health management [10-15 minutes]

1. Would you mind if you and your parent both used an app that focused on playing a game together using an app that was geared toward you taking your medications?
   1. If so:
      1. Would you enjoy trying to solve a puzzle together?
      2. Would you feel burdened about getting prompts or reminders through the app about the game? Or about taking your medication?
      3. Do you mind getting text messages from your parents about medications? Can you describe how your parents currently remind you? What is the system you guys currently use?
      4. How many pings a day from an app would be too much?
      5. Would you mind completing a survey each day about your experience with the app? Or would you prefer weekly?

## Goal 4: Patient stories and “Blue Sky Thinking” activity [~15 minutes]

We will now read you two patient stories about taking a dose of Tacrolimus (or cyclosporine). We ask that each of you think of one creative idea for helping the person in the story to take their dose of Tacrolimus (or cyclosporine). After each of you shares your idea, we will go around again and give you the option to either “keep” the idea you shared, or to “steal” one of the other ideas that you like better. This will help us learn about different ways to help and understand if some ideas appear better than others.

1. **Story 1:** Jordan is a sixteen-year-old. He remembers to take his Tacrolimus (or cyclosporine) on weekdays, but usually forgets to take his doses during the weekend. During the weekends, Jordan is usually online till late because his friends can stay up and play with him online. After a long night of gaming, Jordan is tired and ends up waking up later in the day.
   *What is a creative idea you have for Jordan to help him with taking his Tacrolimus (or cyclosporine)?*
   *(after everyone shares) Would you like to keep your idea or steal one of the other ideas?*
2. **Story 2:** Sam is a seventeen-year-old. She is a senior in high school and feels lonely because she has been unable to participate in clubs she loves in school. She feels as if her mother is always around and asking her about taking her dose. When she takes Tacrolimus (or cyclosporine), it makes her feel ill and if her mother doesn’t remind her, Sam will sometimes skip a dose on purpose to forget about being sick and avoid feeling ill.
   *What is a creative idea you have for Sam to help her with taking her Tacrolimus (or cyclosporine)?
   (after everyone shares) Would you like to keep your idea or steal one of the other ideas?*

## Conclusion [~5 minutes]

Thank you and opportunity to share some final comments and ideas.

# ADAPTS-HCT Focus group session 2 protocol [AYA version]

## Introduction

We are developing a new application to help adolescents and young adults (“AYA”) with taking Tacrolimus (or cyclosporine) during the early phase of transplant. We are focusing primarily on the first 100 days of transplant. The application is designed to be used by both the AYA and the parent/caregiver by providing prompts to help the AYA and their parent/caregiver with the challenges of taking Tacrolimus (or cyclosporine) consistently and receive additional support when needed. The application is NOT an alarm or reminder system – it is aimed to provide help and encouragement that can promote effective habits and routines related to taking this medication.

You can help with the development of this application by sharing your experiences with taking Tacrolimus (or cyclosporine). The purpose of the focus group is to gather feedback about how you remember to take your doses of Tacrolimus (or cyclosporine), the challenges in doing so consistently and how your parent/caregiver supports you in doing so.

We will audio-record the focus group today. This will allow us to go back and listen carefully and make sure that we correctly capture what you said. We will also take notes, but these notes will not be identified by your name or other identifiers. The electronic files are also confidential and will not be shared with people outside of our research team. Some of the topics we talk about might be sensitive, you can skip any question if you feel uncomfortable answering. You can choose not to continue participation at any time during the focus group.

## Goal 1: Current adherence and medication management strategies and challenges [10-15 minutes]

1. Adhering consistently to taking Tacrolimus (or cyclosporine) can be a struggle for AYA.
   1. What are examples of reasons for you missing a dose?
   2. Is there a time of day during which you are more likely to miss a dose?
   3. Can you describe a day during which you were more likely to miss a dose (For example, days of the week; Events that were planned for the day; The way you felt during the day; Slept in too late).
   4. Do interactions with parents/caregivers affect the likelihood of missing a dose? How so?
   5. What do you do if you miss a dose?
      1. Who do you tell?
      2. How do you feel about missing a dose?
   6. Are there things about taking Tacrolimus (or cyclosporine) that you would want to know more about?
   7. What motivates you to keep taking your doses of Tacrolimus (or cyclosporine)?
   8. Are there other health and mental health topics other than Tacrolimus (or cyclosporine) that are important to you?
2. **What strategies do you use to help remember to take your dose of Tacrolimus (or cyclosporine)?**
3. Is there someone that helps you remember to take your dose?
   1. How do you feel about having someone help you remember to take your dose?
   2. Do you prefer to receive help or not?

## Goal 2: Attitudes toward using technology to help taking medication and health management [~15 minutes]

1. Do you use any technology to help remember to take your dose? Do you set up alarms? Do you use an application on your phone? Do you keep a journal/diary to keep track?
2. Using apps and technology for health is becoming more common - do you use any apps to help with managing your health? For example, Fitbit, Apple Health, Samsung Health, or other applications?
   1. If so:
      1. Which do you use?
      2. How does it help you manage your health?
      3. Can you share an example of how it helps you manage your health?
   2. If not:
      1. Have you tried such an app in the past? If so, why did you stop?
      2. Is there a reason you do not use an app to help you manage your health?
3. How do you feel about using an app to support you in developing effective habits and routines for taking your dose consistently and keep track of the doses that you took?
4. How do you feel about your caregiver/parent also using the application to remember to take your dose?
5. Sometimes there is a need to skip a dose of Tacrolimus (or cyclosporine):
   1. How do you know when to skip a dose?
   2. How do you keep track and remember when to take a dose, and when to skip it?
   3. How do you feel about needing to skip a dose?
   4. Is it difficult to remember to take a dose once you are no longer skipping a dose?
   5. How do you communicate skipping a dose with your parent/caregiver?
6. There is new technology that helps keep a record of when you take a dose – for example a special pill bottle with a cap that records anytime you twist it open. Would you want to use such a pill bottle to actively keep track of when you took your dose?
   1. If so, why?
   2. If not, why?
7. We are considering including in the app a function that involves you and your parent collaborating on solving a puzzle game in a way that is geared toward you taking your medications:
   1. How do you feel about trying to solve a simple puzzle together with your parent?
   2. Are you able to find a few minutes to try and solve a puzzle game?
   3. Would it be preferable that each (i.e., you and your parent) solve part of the puzzle separately? Or together? Why?
   4. How often should an app such as this prompt you with messages and about the puzzle? How many prompts a day would be burdensome? How many would be too few?
   5. How do you feel about answering a single question about how you feel when interacting with the app?
   6. How do you feel about answering a survey about your experience with the app on a weekly basis?

## Goal 3: Patient stories and “Blue Sky Thinking” activity [~15 minutes]

We will now read you two patient stories about taking a dose of Tacrolimus (or cyclosporine). We ask that each of you think of one creative idea for helping the person in the story to take their dose of Tacrolimus (or cyclosporine). After each of you shares your idea, we will go around again and give you the option to either “keep” the idea you shared, or to “steal” one of the other ideas that you like better. This will help us learn about different ways to help and understand if some ideas appear better than others.

1. **Story 1:** Jordan is a sixteen-year-old. He remembers to take his Tacrolimus (or cyclosporine) on weekdays, but usually forgets to take his doses during the weekend. During the weekends, Jordan is usually online till late because his friends can stay up and play with him online. After a long night of gaming, Jordan is tired and ends up waking up later in the day.
   *What is a creative idea you have for Jordan to help him with taking his Tacrolimus (or cyclosporine)?*
   *(after everyone shares) Would you like to keep your idea or steal one of the other ideas?*
2. **Story 2:** Sam is a seventeen-year-old. She is a senior in high school and feels lonely because she has been unable to participate in clubs she loves in school. She feels as if her mother is always around and asking her about taking her dose. When she takes Tacrolimus (or cyclosporine), it makes her feel ill and if her mother doesn’t remind her, Sam will sometimes skip a dose on purpose to forget about being sick and avoid feeling ill.
   *What is a creative idea you have for Sam to help her with taking her Tacrolimus (or cyclosporine)?
   (after everyone shares) Would you like to keep your idea or steal one of the other ideas?*

## Conclusion [~5 minutes]

Thank you and opportunity to share some final comments and ideas.

# ADAPTS-HCT Focus group session 2 protocol [Parent version]

## Introduction

We are developing a new application to help adolescents and young adults (“AYA”) with taking Tacrolimus (or cyclosporine) during the early phase of transplant. We are focusing primarily on the first 100 days of transplant. The application is designed to be used by both the AYA and the parent/caregiver by providing prompts to help the AYA and their parent/caregiver with the challenges of taking Tacrolimus (or cyclosporine) consistently and receive additional support when needed. The application is NOT an alarm or reminder system – it is aimed to provide help and encouragement that can promote effective habits and routines related to taking this medication.

You can help with the development of this application by sharing your experiences with supporting your AYA with taking Tacrolimus (or cyclosporine). The purpose of the focus group is to gather feedback about how you interact and support your AYA with remembering to take their doses of Tacrolimus (or cyclosporine), the challenges in doing so consistently, and how this affects you as a parent/caregiver.

We will audio-record the focus group today. This will allow us to go back and listen carefully and make sure that we correctly capture what you said. We will also take notes, but these notes will not be identified by your name or other identifiers. The electronic files are also confidential and will not be shared with people outside of our research team. Some of the topics we talk about might be sensitive, you can skip any question if you feel uncomfortable answering. You can choose not to continue participation at any time during the focus group.

## Goal 1: Current adherence and medication management strategies and challenges [10-15 minutes]

1. Adhering consistently to taking Tacrolimus (or cyclosporine) can be a struggle for AYA.
   1. What are examples of reasons for your AYA missing a dose?
   2. Is there a time of day during which your AYA is more likely to miss a dose?
   3. Can you describe a day during which your AYA was more likely to miss a dose (For example, days of the week; Events that were planned for the day; The way the AYA felt during the day; Slept in too late).
   4. Do interactions with you (parents/caregiver) affect the likelihood of your AYA missing a dose? How so?
   5. What do you do if your AYA misses a dose?
      1. Who do you tell?
      2. How do you feel about your AYA missing a dose?
   6. Are there things about taking Tacrolimus (or cyclosporine) that you would want to know more about?
   7. What motivates you to keep supporting your AYA in taking their doses of Tacrolimus (or cyclosporine)?
   8. Are there other health and mental health topics other than Tacrolimus (or cyclosporine) that are important to you?
2. **What strategies do you use to help your AYA remember to take their dose of Tacrolimus (or cyclosporine)?**
3. Do you play an important role in the AYA remembering to take their doses?
   1. How do you feel about having to help the AYA remember to take their dose?
   2. Do you prefer not to provide help or not?

## Goal 2: Attitudes toward using technology to help taking medication and health management [~15 minutes]

1. Do you use any technology to help the AYA remember to take their dose? Do you set up alarms? Do you use an application on your phone? Do you keep a journal/diary to keep track?
2. Using apps and technology for health is becoming more common - do you use any apps to help with managing your own health? For example, Fitbit, Apple Health, Samsung Health, or other applications?
   1. If so:
      1. Which do you use?
      2. How does it help you manage your health?
      3. Can you share an example of how it helps you manage your health?
   2. If not:
      1. Have you tried such an app in the past? If so, why did you stop?
      2. Is there a reason you do not use an app to help you manage your health?
3. How do you feel about using an app to help the AYA remember to take their dose and keep track of the doses that they took?
4. How do you feel about you (as the caregiver/parent) also using the same application to help the AYA remember to take their dose?
5. Sometimes there is a need to skip a dose of Tacrolimus (or cyclosporine):
   1. How do you know when your AYA needs to skip a dose?
   2. How do you keep track and remember when the AYA needs to take a dose, and when to skip it?
   3. How do you feel about your AYA needing to skip a dose?
   4. Is it difficult to keep track of when your AYA needs to take a dose once they are no longer skipping a dose?
   5. How does your AYA communicate skipping a dose with you?
6. There is new technology that helps keep a record of when a dose is taken – for example a special pill bottle with a cap that records anytime it is twisted open. Would you want to use such a pill bottle to actively keep track of when your AYA took their dose?
   1. If so, why?
   2. If not, why?
7. We are considering including in the app a function that involves you and your AYA collaborating on solving a puzzle game in a way that is geared toward the AYA taking their medications:
   1. How do you feel about trying to solve a simple puzzle together with your AYA?
   2. Are you able to find a few minutes to try and solve a puzzle game?
   3. Would it be preferable that each (i.e., you and the AYA) solve part of the puzzle separately? Or together? Why?
   4. How often should an app such as this prompt you with messages and about the puzzle? How many prompts a day would be burdensome? How many would be too few?
   5. How do you feel about answering a single question about how you feel when interacting with the app?
   6. How do you feel about answering a survey about your experience with the app on a weekly basis?

## Goal 3: Patient stories and “Blue Sky Thinking” activity [~15 minutes]

We will now read you two patient stories about taking a dose of Tacrolimus (or cyclosporine). We ask that each of you think of one creative idea for helping the person in the story to take their dose of Tacrolimus (or cyclosporine). After each of you shares your idea, we will go around again and give you the option to either “keep” the idea you shared, or to “steal” one of the other ideas that you like better. This will help us learn about different ways to help and understand if some ideas appear better than others.

1. **Story 1:** Jordan is a sixteen-year-old. He remembers to take his Tacrolimus (or cyclosporine) on weekdays, but usually forgets to take his doses during the weekend. During the weekends, Jordan is usually online till late because his friends can stay up and play with him online. After a long night of gaming, Jordan is tired and ends up waking up later in the day.
   *What is a creative idea you have for Jordan to help him with taking his Tacrolimus (or cyclosporine)?*
   *(after everyone shares) Would you like to keep your idea or steal one of the other ideas?*
2. **Story 2:** Sam is a seventeen-year-old. She is a senior in high school and feels lonely because she has been unable to participate in clubs she loves in school. She feels as if her mother is always around and asking her about taking her dose. When she takes Tacrolimus (or cyclosporine), it makes her feel ill and if her mother doesn’t remind her, Sam will sometimes skip a dose on purpose to forget about being sick and avoid feeling ill.
   *What is a creative idea you have for Sam to help her with taking her Tacrolimus (or cyclosporine)?
   (after everyone shares) Would you like to keep your idea or steal one of the other ideas?*

## Conclusion [~5 minutes]

Thank you and opportunity to share some final comments and ideas.

# ADAPTS-HCT mock up interview 1 protocol [AYA version]

## Introduction

We are developing a new application to help adolescents and young adults (“AYA”) with taking Tacrolimus (or cyclosporine) during the early phase of transplant. We are focusing primarily on the first 100 days of transplant. The application is designed to be used by both the AYA and the parent/caregiver by providing prompts to help the AYA and their parent/caregiver with taking Tacrolimus (or cyclosporine) consistently and receive additional support when needed.

You can help with the development of this application by sharing your experiences with taking Tacrolimus (or cyclosporine). The purpose of the focus group is to gather feedback about how you remember to take your doses of Tacrolimus (or cyclosporine) and how your parent/caregiver supports you in doing so.

We will also show you a few mock up sketches of what this app might look like – we will ask you to “think aloud” as you see various screen sketches – this means to describe in your words what thoughts you have as you look at the screen sketch and share what your thoughts are about tapping and what you expect might happen. For some elements in the screen sketches, we will have additional screen sketches to show you – similar to what would happen if this was a real app. The more you can share about your thoughts about the screen sketches, the more we can use that to build the actual app (as we complete this we will share with you if you are interested).

We will audio-record the focus group today. This will allow us to go back and listen carefully and make sure that we correctly capture what you said. We will also take notes, but these notes will not be identified by your name or other identifiers. The electronic files are also confidential and will not be shared with people outside of our research team. Some of the topics we talk about might be sensitive, you can skip any question if you feel uncomfortable answering. You can choose not to continue participation at any time during the focus group.

## Goal 1: Current adherence and medication management strategies and challenges [5-10 minutes]

1. Adhering consistently to taking Tacrolimus (or cyclosporine) can be a struggle for AYA.
2. What are examples of reasons for you missing a dose?
3. Is there a time of day during which you are more likely to miss a dose?
4. Can you describe a day during which you were more likely to miss a dose of (For example, days of the week; Events that were planned for the day; The way you felt during the day; Slept in too late).
5. Do interactions with parents/caregivers affect the likelihood of missing a dose? How so?
6. What do you do if you miss a dose?
   1. Who do you tell?
   2. How do you feel about missing a dose?
7. Are there things about taking Tacrolimus (or cyclosporine) that you would want to know more about?
8. What motivates you to keep taking your doses of Tacrolimus (or cyclosporine)?
9. Are there other health and mental health topics other than Tacrolimus (or cyclosporine) that are important to you?
10. **What strategies do you use to help remember to take your dose of Tacrolimus (or cyclosporine)?**
11. Is there someone that helps you remember to take your dose?
12. How do you feel about having someone help you remember to take your dose?
13. Do you prefer not to receive help or not?

## Goal 2: Attitudes toward using technology to help taking medication and health management [5-10 minutes]

1. Do you use any technology to help remember to take your dose? Do you set up alarms? Do you use an application on your phone? Do you keep a journal/diary to keep track?
2. Using apps and technology for health is becoming more common:
3. Do you use any apps to help with managing your health? For example, Fitbit, Apple Health, Samsung Health, or other applications?
   1. If so:
      1. Which do you use?
      2. How does it help you manage your health?
      3. Can you share an example of how it helps you manage your health?
   2. If not:
      1. Have you tried such an app in the past? If so, why did you stop?
      2. Is there a reason you do not use an app to help you manage your health?
4. How do you feel about using an app to help you remember to take your dose, and keep track of the doses that you took?
5. How do you feel about your caregiver/parent also using the application to help you remember to take your dose?
6. Sometimes there is a need to skip a dose of Tacrolimus (or cyclosporine):
7. How do you know when to skip a dose?
8. How do you keep track and remember when to take a dose, and when to skip it?
9. How do you feel about needing to skip a dose?
10. Is it difficult to remember to take a dose once you are no longer skipping a dose?
11. How do you communicate skipping a dose with your parent/caregiver?

## Goal 3: Attitudes about your parents helping you with your medication and health management [5-10 minutes]

1. Would you mind if you and your parent both used an app that focused on playing a game together using an app that was geared toward you taking your medications?
   1. If so:
      1. Would you enjoy trying to solve a puzzle together?
      2. Would you feel burdened about getting prompts or reminders through the app about the game? Or about taking your medication?
      3. Do you mind getting text messages from your parents about medications? Can you describe how your parents currently remind you? What is the system you guys currently use?
      4. How many pings a day from an app would be too much?
      5. Would you mind completing a survey each day about your experience with the app? Or would you prefer weekly?

## Goal 4: screen mockup think aloud procedure – 20-25 minutes – see PowerPoint.

## Conclusion [~5 minutes]

Thank you and opportunity to share some final comments and ideas.

## General guidelines for think aloud procedure:

Review the notes in the power point (each slide has notes added – make sure you can view these).

There are three sections in the file: (a) AYA medication logging (reporting); (b) Parent view of medication log (parents can only view, so does not include editing and reason functions) – this makes the parent script much shorter; and (c) game demo – both parent and AYA will see the game from the perspective of the AYA (difference is only in the indicator around the assigned words).

Each slide is labeled in top left corner – these slide numbers are referenced in the notes to help you how to find the order in which to show slides based on what the parent or AYA say they are tapping (notes list this at the end of each page).

You can start with reading the following paragraph (it is in the introduction too):

*As we mentioned at the start, we will now show you a few mock up sketches of what this app might look like – we will ask you to “think aloud” as you see various screen sketches – this means to describe in your words what thoughts you have as you look at the screen sketch and share what your thoughts are about tapping and what you expect might happen. For some elements in the screen sketches, we will have additional screen sketches to show you – similar to what would happen if this was a real app. The more you can share about your thoughts about the screen sketches, the more we can use that to build the actual app (as we complete this we will share the app with you if you are interested).*

When you show each slide in the script from the PPT, use language such as:

- Imagine you just received this notification on your phone – look at this, can you describe your thoughts as you think about what to do next?
- Is there anything you would do with this screen?
- If asked about how to use it – imagine this was an app on your phone – how do you do things with an app? If still stuck, use language such as tapping is usually something we do with an app on our phone – is there something here that you would tap? Why? What would you expect would happen?
- If they try to do something that is off script and/or not implemented – tell them you don’t have a sketch for that, but please describe what you would expect it to show? Why?
- If something is not supposed to react to tapping – you can say, “nothing happens – what do you do next?”
- As each screen is presented, use language such as: this is what shows up after you did X. What are your thoughts as you see this? Is there anything you do?
- If stuck on a certain part of the script and short on time, use language to move to the next segment in the script, for example: your experience and reaction to this set of screens is really helpful and important as we develop an app – we need to move on to the next screen to show you so that we can learn from you about more parts of the app.
- When discussing a screen make sure that you say the slide number and slide number subtitle so that we can connect the transcript to what they are seeing.

# ADAPTS-HCT mock up interview 1 protocol [Parent version]

## Introduction

We are developing a new application to help adolescents and young adults (“AYA”) with taking Tacrolimus (or cyclosporine) during the early phase of transplant. We are focusing primarily on the first 100 days of transplant. The application is designed to be used by both the AYA and the parent/caregiver by providing prompts to help the AYA and their parent/caregiver with taking Tacrolimus (or cyclosporine) consistently and receive additional support when needed.

You can help with the development of this application by sharing your experiences with supporting your AYA with taking Tacrolimus (or cyclosporine). The purpose of the focus group is to gather feedback about how you interact and support your AYA with remembering to take their doses of Tacrolimus (or cyclosporine).

We will also show you a few mock up sketches of what this app might look like – we will ask you to “think aloud” as you see various screen sketches – this means to describe in your words what thoughts you have as you look at the screen sketch and share what your thoughts are about tapping and what you expect might happen. For some elements in the screen sketches, we will have additional screen sketches to show you – similar to what would happen if this was a real app. The more you can share about your thoughts about the screen sketches, the more we can use that to build the actual app (as we complete this we will share with you if you are interested).

We will audio-record the focus group today. This will allow us to go back and listen carefully and make sure that we correctly capture what you said. We will also take notes, but these notes will not be identified by your name or other identifiers. The electronic files are also confidential and will not be shared with people outside of our research team. Some of the topics we talk about might be sensitive, you can skip any question if you feel uncomfortable answering. You can choose not to continue participation at any time during the focus group.

## Goal 1: Current adherence and medication management strategies and challenges [5-10 minutes]

1. Adhering consistently to taking Tacrolimus (or cyclosporine) can be a struggle for AYA.
2. What are examples of reasons for your AYA missing a dose?
3. Is there a time of day during which your AYA is more likely to miss a dose?
4. Can you describe a day during which your AYA was more likely to miss a dose (For example, days of the week; Events that were planned for the day; The way the AYA felt during the day; Slept in too late).
5. Do interactions with you (parents/caregiver) affect the likelihood of your AYA missing a dose? How so?
6. What do you do if your AYA misses a dose?
   1. Who do you tell?
   2. How do you feel about your AYA missing a dose?
7. Are there things about taking Tacrolimus (or cyclosporine) that you would want to know more about?
8. What motivates you to keep supporting your AYA in taking their doses of Tacrolimus (or cyclosporine)?
9. Are there other health and mental health topics other than Tacrolimus (or cyclosporine) that are important to you?
10. **What strategies do you use to help your AYA remember to take their dose of Tacrolimus (or cyclosporine)?**
11. Do you play an important role in the AYA remembering to take their doses?
12. How do you feel about having to help the AYA remember to take their dose?
13. Do you prefer not to provide help or not?

## Goal 2: Attitudes toward using technology to help taking medication and health management [5-10 minutes]

1. Do you use any technology to help the AYA remember to take their dose? Do you set up alarms? Do you use an application on your phone? Do you keep a journal/diary to keep track?
2. Using apps and technology for health is becoming more common:
3. Do you use any apps to help with managing your own health? For example, Fitbit, Apple Health, Samsung Health, or other applications?
   1. If so:
      1. Which do you use?
      2. How does it help you manage your health?
      3. Can you share an example of how it helps you manage your health?
   2. If not:
      1. Have you tried such an app in the past? If so, why did you stop?
      2. Is there a reason you do not use an app to help you manage your health?
4. How do you feel about using an app to help the AYA remember to take their dose and keep track of the doses that they took?
5. How do you feel about you (as the caregiver/parent) also using the same application to help the AYA remember to take their dose?
6. Sometimes there is a need to skip a dose of Tacrolimus (or cyclosporine):
7. How do you know when your AYA needs to skip a dose?
8. How do you keep track and remember when the AYA needs to take a dose, and when to skip it?
9. How do you feel about your AYA needing to skip a dose?
10. Is it difficult to keep track of when your AYA needs to take a dose once they are no longer skipping a dose?
11. How does your AYA communicate skipping a dose with you?

## Goal 3: Attitudes about your parents helping you with your medication and health management [5-10 minutes]

1. Would you mind if you and your parent both used an app that focused on playing a game together using an app that was geared toward you taking your medications?
   1. If so:
      1. Would you enjoy trying to solve a puzzle together?
      2. Would you feel burdened about getting prompts or reminders through the app about the game? Or about taking your medication?
      3. Do you mind getting text messages from your parents about medications? Can you describe how your parents currently remind you? What is the system you guys currently use?
      4. How many pings a day from an app would be too much?
      5. Would you mind completing a survey each day about your experience with the app? Or would you prefer weekly?

## Goal 4: screen mockup think aloud procedure –15-20 minutes – see PowerPoint (parent script is shorter than that for AYA).

## Conclusion [~5 minutes]

Thank you and opportunity to share some final comments and ideas.

## General guidelines for think aloud procedure:

Review the notes in the power point (each slide has notes added – make sure you can view these).

There are three sections in the file: (a) AYA medication logging (reporting); (b) Parent view of medication log (parents can only view, so does not include editing and reason functions) – this makes the parent script much shorter; and (c) game demo – both parent and AYA will see the game from the perspective of the AYA (difference is only in the indicator around the assigned words).

Each slide is labeled in top left corner – these slide numbers are referenced in the notes to help you how to find the order in which to show slides based on what the parent or AYA say they are tapping (notes list this at the end of each page).

You can start with reading the following paragraph (it is in the introduction too):

*As we mentioned at the start, we will now show you a few mock up sketches of what this app might look like – we will ask you to “think aloud” as you see various screen sketches – this means to describe in your words what thoughts you have as you look at the screen sketch and share what your thoughts are about tapping and what you expect might happen. For some elements in the screen sketches, we will have additional screen sketches to show you – similar to what would happen if this was a real app. The more you can share about your thoughts about the screen sketches, the more we can use that to build the actual app (as we complete this we will share the app with you if you are interested).*

When you show each slide in the script from the PPT, use language such as:

- Imagine you just received this notification on your phone – look at this, can you describe your thoughts as you think about what to do next?
- Is there anything you would do with this screen?
- If asked about how to use it – imagine this was an app on your phone – how do you do things with an app? If still stuck, use language such as tapping is usually something we do with an app on our phone – is there something here that you would tap? Why? What would you expect would happen?
- If they try to do something that is off script and/or not implemented – tell them you don’t have a sketch for that, but please describe what you would expect it to show? Why?
- If something is not supposed to react to tapping – you can say, “nothing happens – what do you do next?”
- As each screen is presented, use language such as: this is what shows up after you did X. What are your thoughts as you see this? Is there anything you do?
- If stuck on a certain part of the script and short on time, use language to move to the next segment in the script, for example: your experience and reaction to this set of screens is really helpful and important as we develop an app – we need to move on to the next screen to show you so that we can learn from you about more parts of the app.
- When discussing a screen make sure that you say the slide number and slide number subtitle so that we can connect the transcript to what they are seeing.

# ADAPTS-HCT mockup interview 2 protocol [AYA version]

## Introduction

We are developing a new application to help adolescents and young adults (“AYA”) with taking Tacrolimus (or cyclosporine) during the early phase of transplant. We are focusing primarily on the first 100 days of transplant. The application is designed to be used by both the AYA and the parent/caregiver by providing prompts to help the AYA and their parent/caregiver with taking Tacrolimus (or cyclosporine) consistently and receive additional support when needed.

You can help with the development of this application by sharing your experiences with taking medication that also requires a strict adherence to taking a first daily dose in the morning and a second daily dose in the evening (for example, Tacrolimus or cyclosporine). Keep in mind that an app that helps keep track of ALL medications and their doses is not currently within the scope we are working on in this study. The purpose of this interview is to gather feedback about how you remember to take your doses of medications with this type of schedule, such as Tacrolimus (or cyclosporine) and how your parent/caregiver supports you in doing so.

We will also show you a few mockup sketches of what this app might look like – we will ask you to “think aloud” as you see various screen sketches – this means to describe in your words what thoughts you have as you look at the screen sketch and share what your thoughts are about tapping and what you expect might happen. For some elements in the screen sketches, we will have additional screen sketches to show you – similar to what would happen if this was a real app. The more you can share about your thoughts about the screen sketches, the more we can use that to build the actual app (as we complete this we will share with you if you are interested).

We will audio-record the interview, and if possible, record video (only) of the sketches we share with you today. This will allow us to go back and listen carefully and make sure that we correctly capture what you said and what sketches were displayed as you spoke. We will also take notes, but these notes will not be identified by your name or other identifiers. The electronic files are also confidential and will not be shared with people outside of our research team. Some of the topics we talk about might be sensitive, you can skip any question if you feel uncomfortable answering. You can choose not to continue participation at any time during the focus group.

## Goal 1: Current adherence and medication management strategies and challenges [5-10 minutes]

1. Adhering consistently to taking Tacrolimus (or cyclosporine) can be a struggle for AYA.
2. What are examples of reasons for you missing a dose?
3. Is there a time of day during which you are more likely to miss a dose?
4. **What strategies do you use to help remember to take your dose of Tacrolimus (or cyclosporine)?**

## Goal 2: Attitudes toward using technology to help taking medication and health management [10 minutes]

1. How do you feel about using an app to help you remember to take your dose, and keep track of the doses that you took?
2. How do you feel about your caregiver/parent also using the same application to help you remember to take your dose?
3. Would you mind if you and your parent both used an app that focused on keeping a log of taking a specific medication dose in the morning and the evening, and also sometimes incorporated playing a game together using an app that was geared toward supporting you and your parent with the challenges of adhering to taking such a medication?
   1. If so:
      1. Would you enjoy trying to solve a puzzle together?
      2. Would you feel burdened about getting prompts or reminders through the app about the game? Or about taking your medication?
      3. Would you mind completing a survey about your experience with the app? What would be a reasonable frequency for being asked to fill in such a survey?

## Goal 3: screen mockup think aloud procedure – 20-25 minutes – see PowerPoint.

## Conclusion [~5 minutes]

Thank you and opportunity to share some final comments and ideas.

## General guidelines for think aloud procedure:

Review the notes in the power point (each slide has notes added – make sure you can view these).

There are three sections in the file: (a) AYA medication logging (reporting); (b) Parent view of medication log (parents can only view, so does not include editing and reason functions) – this makes the parent script much shorter; and (c) game demo – both parent and AYA will see the game from the perspective of the AYA (difference is only in the indicator around the assigned words).

Each slide is labeled in top left corner – these slide numbers are referenced in the notes to help you how to find the order in which to show slides based on what the parent or AYA say they are tapping (notes list this at the end of each page).

You can start with reading the following paragraph (it is in the introduction too):

*As we mentioned at the start, we will now show you a few mock up sketches of what this app might look like – we will ask you to “think aloud” as you see various screen sketches – this means to describe in your words what thoughts you have as you look at the screen sketch and share what your thoughts are about tapping and what you expect might happen. For some elements in the screen sketches, we will have additional screen sketches to show you – similar to what would happen if this was a real app. The more you can share about your thoughts about the screen sketches, the more we can use that to build the actual app (as we complete this we will share the app with you if you are interested).*

When you show each slide in the script from the PPT, use language such as:

- Imagine you just received this notification on your phone – look at this, can you describe your thoughts as you think about what to do next?
- Is there anything you would do with this screen?
- If asked about how to use it – imagine this was an app on your phone – how do you do things with an app? If still stuck, use language such as tapping is usually something we do with an app on our phone – is there something here that you would tap? Why? What would you expect would happen?
- If they try to do something that is off script and/or not implemented – tell them you don’t have a sketch for that, but please describe what you would expect it to show? Why?
- If something is not supposed to react to tapping – you can say, “nothing happens – what do you do next?”
- As each screen is presented, use language such as: this is what shows up after you did X. What are your thoughts as you see this? Is there anything you do?
- If stuck on a certain part of the script and short on time, use language to move to the next segment in the script, for example: your experience and reaction to this set of screens is really helpful and important as we develop an app – we need to move on to the next screen to show you so that we can learn from you about more parts of the app.
- When discussing a screen make sure that you say the slide number and slide number subtitle so that we can connect the transcript to what they are seeing.

# ADAPTS-HCT mockup interview 2 protocol [Parent version]

## Introduction

We are developing a new application to help adolescents and young adults (“AYA”) with taking Tacrolimus (or cyclosporine) during the early phase of transplant. We are focusing primarily on the first 100 days of transplant. The application is designed to be used by both the AYA and the parent/caregiver by providing prompts to help the AYA and their parent/caregiver with taking Tacrolimus (or cyclosporine) consistently and receive additional support when needed.

You can help with the development of this application by sharing your experiences with supporting your AYA with taking medication that also requires strict adherence to taking a dose in the morning and a second dose in the evening (for example, Tacrolimus or cyclosporine). Keep in mind that an app that helps keep track of ALL medications and their doses is not currently within the scope we are working on in this study. The purpose of this interview is to gather feedback about how you interact and support your AYA with remembering to take their doses of medications with this type of schedule, such as Tacrolimus (or cyclosporine).

We will also show you a few mockup sketches of what this app might look like – we will ask you to “think aloud” as you see various screen sketches – this means to describe in your words what thoughts you have as you look at the screen sketch and share what your thoughts are about tapping and what you expect might happen. For some elements in the screen sketches, we will have additional screen sketches to show you – similar to what would happen if this was a real app. The more you can share about your thoughts about the screen sketches, the more we can use that to build the actual app (as we complete this we will share with you if you are interested).

We will audio-record the interview, and if possible, record video (only) of the sketches we share with you today. This will allow us to go back and listen carefully and make sure that we correctly capture what you said and what sketches were displayed as you spoke. We will also take notes, but these notes will not be identified by your name or other identifiers. The electronic files are also confidential and will not be shared with people outside of our research team. Some of the topics we talk about might be sensitive, you can skip any question if you feel uncomfortable answering. You can choose not to continue participation at any time during the focus group.

## Goal 1: Current adherence and medication management strategies and challenges [5 minutes]

1. Adhering consistently to taking Tacrolimus (or cyclosporine) can be a struggle for AYA.
2. What are examples of reasons for your AYA missing a dose?
3. Is there a time of day during which your AYA is more likely to miss a dose?
4. **What strategies do you use to help your AYA remember to take their dose of Tacrolimus (or cyclosporine)?**

## Goal 2: Attitudes toward using technology to help taking medication and health management [10 minutes]

1. How do you feel about using an app to help the AYA remember to take their dose and keep track of the doses that they took?
2. How do you feel about you (as the caregiver/parent) also using the same application to help the AYA remember to take their dose?
3. How do you feel about the AYA using an activity tracker such as Fitbit to allow the app to better adapt to the AYA’s needs.
4. Would you mind if you and your AYA both used an app that focused on keeping a log of taking a specific medication dose in the morning and the evening, and also sometimes incorporated playing a game together using an app that was geared toward supporting you and your AYA’s challenges with adhering to taking such a medication?
   1. If so:
      1. Would you enjoy trying to solve a puzzle together?
      2. Would you feel burdened about getting prompts or reminders through the app about the game? (For AYA: Or about taking your medication?)
      3. Would you mind completing a survey about your experience with the app? What would be a reasonable frequency for being asked to fill in such a survey?

## Goal 3: screen mockup think aloud procedure –15-20 minutes – see PowerPoint (parent script is shorter than that for AYA).

## Conclusion [~5 minutes]

Thank you and opportunity to share some final comments and ideas.

## General guidelines for think aloud procedure:

Review the notes in the power point (each slide has notes added – make sure you can view these).

There are three sections in the file: (a) AYA medication logging (reporting); (b) Parent view of medication log (parents can only view, so does not include editing and reason functions) – this makes the parent script much shorter; and (c) game demo – both parent and AYA will see the game from the perspective of the AYA (difference is only in the indicator around the assigned words).

Each slide is labeled in top left corner – these slide numbers are referenced in the notes to help you how to find the order in which to show slides based on what the parent or AYA say they are tapping (notes list this at the end of each page).

You can start with reading the following paragraph (it is in the introduction too):

*As we mentioned at the start, we will now show you a few mock up sketches of what this app might look like – we will ask you to “think aloud” as you see various screen sketches – this means to describe in your words what thoughts you have as you look at the screen sketch and share what your thoughts are about tapping and what you expect might happen. For some elements in the screen sketches, we will have additional screen sketches to show you – similar to what would happen if this was a real app. The more you can share about your thoughts about the screen sketches, the more we can use that to build the actual app (as we complete this we will share the app with you if you are interested).*

When you show each slide in the script from the PPT, use language such as:

- Imagine you just received this notification on your phone – look at this, can you describe your thoughts as you think about what to do next?
- Is there anything you would do with this screen?
- If asked about how to use it – imagine this was an app on your phone – how do you do things with an app? If still stuck, use language such as tapping is usually something we do with an app on our phone – is there something here that you would tap? Why? What would you expect would happen?
- If they try to do something that is off script and/or not implemented – tell them you don’t have a sketch for that, but please describe what you would expect it to show? Why?
- If something is not supposed to react to tapping – you can say, “nothing happens – what do you do next?”
- As each screen is presented, use language such as: this is what shows up after you did X. What are your thoughts as you see this? Is there anything you do?
- If stuck on a certain part of the script and short on time, use language to move to the next segment in the script, for example: your experience and reaction to this set of screens is really helpful and important as we develop an app – we need to move on to the next screen to show you so that we can learn from you about more parts of the app.
- When discussing a screen make sure that you say the slide number and slide number subtitle so that we can connect the transcript to what they are seeing.
