# Supplementary material for: Exploring Strategies for a Digital Tool to Support Medication Adherence Among Adolescents and Young Adults Undergoing Hematopoietic Stem Cell Transplant and Their Care Partners: Qualitative Formative Study
Source: JMIR Form Res. 2026 Feb 17;10:e82356. doi: 10.2196/82356 (PMC12957942; doi:10.2196/82356)
Supplement: Multimedia Appendix 2 [file formative_v10i1e82356_app2.docx]

**Multimedia Appendix II: Medication Adherence Focus Group and Interview Codebook**

Purpose: As formative research for early-stage intervention development, our aims were to: 1) explore current HCT medication adherence strategies and challenges; 2) understand attitudes toward digital technology, including dyadic perspectives on using an app together to support adherence; and 3) assess AYA–care partner relationships, including AYAs’ views on care partner involvement. This process was intended to inform the design of a relevant and user-centered mHealth app.

| **Code Name** | **Code** | **Description** | **Examples** |
| --- | --- | --- | --- |
| **Patient- Reasoning surrounding missing dose** | PT-REAS | PT describes a common reason they miss a dose   - Work - Out with friends - Nighttime - Weekends - Mornings | “One big reason that I miss a dose is sleeping in. I like to sleep in. A lot of the time, I sleep in past 12:00 p.m., so I'll miss that morning dose, but I typically won't miss the evening dose.” |
| **Patient- Feelings/concerns after missing a dose** | PT-CONC | PT describes concerns after missing a dose   - Anxiety - Altered feelings - Lack of concern | “Nervous, I guess, because I know that it’s something that needs to be taken regularly. I’d probably call the clinic and ask what they wanted me to do.” |
| **Patient- Anxiety following missing a dose** | PT-ANX | PT describes why they have anxiety surrounding missing a dose   - Dr. makes it sound scary if dose is missed | “Yes, that’s the other thing. Sometimes the anxiety drives you to keep it a secret and not just take it on your own” |
| **Patient- Time of day missing dose** | PT-TIME | PT explains why at a certain timeframe they are more likely to miss a dose   - Nighttime - Morning sleep in too late | “The morning dose is more challenging?  [Patient]: Most definitely.”  “I think morning.” |
| **Patient- Type of day missing dose** | PT-TYPE | PT gives examples of days he would be more likely to skip a dose   - Weekends, lose structure - Study Days | “Yes, the weekends more, because I want to sleep in more.” |
| **Patient- Inpatient consideration** | PT-INPATIENT | PT describes how being inpatient has different benefits or challenges compared to being outpatient regarding medication adherence   - Routine is monitored more closely | “That’s not as much of a concern right now, because I’m inpatient, but as soon as I’m outpatient, that’s going to be the system I probably have to go to.” |
| **Patient- Caregiver interaction** | PT-CG-INT | PT describes how CG interactions can affect them taking   - Caregiver reminders - Forgetting - Annoyance - Backup | “Yes. The more I interact with my caregiver, which is my husband (laughs), he keeps me on my toes and reminds me, and so I make sure I get up and take it then.” |
| **Patient- Missed dose feelings** | PT-MISSED | PT describes concerns after missing a dose   - No big deal - It adds up | “Maybe a little guilty or unprepared sometimes.” |
| **Patient-Who does patient tell if missed dose** | PT-TELL-MISS | PT explains who they tell if they miss a dose   - Parents - Girlfriend - Doctor | “Usually my parents will be asking me, “Why are you scrambling around looking for your medication?” when I forgot to take it” |
| **Patient-Motivation to keep taking medication** | PT-MOTIVE | PT describes their motivation for taking medication   - Will to live - People pleasing - Anxious - Leaving the hospital | “Yes, full selfish. I want to live.”  “Because I know that it’s helping me recover.” |
| **Patient- Feelings about medications** | PT-FEEL | PT describes any feelings they have about the medication they take   - Normal pill - Not aggressive - Science behind it | “Yes, I’m counting my blessings that the tacro is just a pill, because in the last month or so, I’ve also had to deal with subcutaneous injections, and for the last year, eyedrops.” |
| **Patient- Mental Health concerns/thoughts** | PT-MENT-HTLH | PT describes mental health concerns surrounding their illness   - Survivor’s Guilt - loneliness | “When you have friends who are going through the worst part of their life in their treatment and you’re sitting there having already done it and fine, and it’s not going as well for them as it’s going for you.” |
| **Patient- Adherence Strategies** | PT-STRAT | PT describes the strategies they employ to remember taking medication   - Alarm (most common) - Pill container - Pair with activity | “After a while, I just started putting one thing I do every day with taking my medicine.” |
| **Patient- Who reminds patient to take medication** | PT-REMINDS | PT explains who in their life reminds them to take their medication   - Parents - Colleague - Significant other | “Yes, my husband, my caretaker.” |
| **Patient- Knowledge on medications they are taking** | PT-KNOW | PT explains the type of knowledge they have or would like to have regarding the medications they are currently taking   - “prevents my body from destroying the organ” - Science behind the medicine | “they let me know what my medicine did for me because sickle cell, my blood cells aren’t shaped as an oval, so they restore new blood cells, so I really understood enough.” |
| **Patient- App v. Caregiver preference** | PT-APP-VS-CG | PT describes their feelings towards having someone help them remember to take their medication vs having no help   - Easier to deal with - Sense of autonomy intact | “It’d probably be better if it was an app or an inanimate object yelling at me, rather than my own mom. It’d be easier to deal with.” |
| **Patient- Technology aiding adherence** | PT-TECH | PT describes what technology they use to remember taking medication   - Alarms - Other apps - reminders | “It would just basically set alarms— Not alarms, but like… Everyone nowadays, we’re all on our phones, so in the morning, if I wake up— I think I remember one day I didn’t take it right away, so I looked on my phone and I was scrolling through my messages, notification center, and I saw the notification.” |
| **Patient- mHealth use for overall health/wellness** | PT-MHEALTH | PT describes what technology they use for general health management   - Step tracker - Nike Training App - My fitness pal - Weightlifting- strong app - Pikman Bloom | “I have the Health app on my phone. I use it minimally, but yes” |
| **Patient- feelings on adherence app** | PT-APP-FEEL | PT describes their feelings on using an app to remember to take dose/keep track of dosing   - Not opposed to it | “I would think that would be really helpful, especially if it had some logging system to it to account for times and different things like that.” |
| **Patient- feelings on caregiver use in app** | PT-APP-CG-FEEL | PT gives their feelings on caregiver using the app   - Pushes patient to do better - “watching me like a hawk” - Fine line | “I think there’s a fine line. I think it very much is dependent on the family and the patient and how they interact, because in some families, it’s like, “Hey, I know you didn’t take your medicine today,” and then you’ll take it, and that would be completely fine. Whereas some other people might feel as though, “You’re watching me like a hawk. I haven’t even had a chance to take my medicine yet.” It’s very dependent. I think, in general, it can be helpful to have that little nudge, to be helpful.” |
| **Patient- knowing when to skip dose** | PT-SKIP | PT explains how they know when to skip a dose   - Waits for doctor - Parents | “I’ll just wait for the doctor to tell me, like, “We’re going to hold this medication for today because of X, Y, Z.” That’s really it.” |
| **Patient- How patient keeps track of taking dose** | PT-TRACK | How PT keeps track of taking their dose   - Pill container - Alter schedule | “I have a week container, so Mom will usually just take it out and then I’ll just take my meds like normal.” |
| **Patient- skipping dose feelings** | PT-SKIP-FEEL | PT describes their feelings surrounding needing to skip a dose   - One less pill - Patient is not the medical professional | “It depends on what I’m skipping it for. Sometimes I have to skip a dose to go for blood work, and then sometimes I just have to skip a dose because that's what the medicine calls for. I feel pretty fine about skipping doses. It's just when I miss the dose, I get a little concerned, ‘Oh, man.’” |
| **Patient- skipping dose difficulties** | PT-SKIP-DIFF | PT describes the difficulties surrounding needing to skip a dose   - Difficult to get back into routine | “I don’t think it’s difficult to start taking it again.”  “It could be if I forget to turn back on the reminders or go back into routine, but I feel like most people would have a hard time getting back to routine but will get back to it quickly if they’re used to it.” |
| **Patient- communication in dyad** | PT-DYAD-COMM | How patient would communicate skipping a dose with CG   - Mistake - Forgot it - Always tell Dr. | “I just tell them that I made a mistake and forgot it.”  “I don’t know that I would necessarily communicate it to my caregiver, but that’s just me” |
| **Patient- special pill bottle opinions** | PT-ECAP | PT describes their thoughts on if they would want to use a new technology, special pill bottle   - Pez dispenser - Easily cheese eCap - Bad for weekly pill containers | “No, because sometimes the changing of the doses and them already being preset, I have to go in and re-change and reset it up. No. I've used a lot of different pill box types too, so maybe the digital would be a little better, but it might be a little too much or a little too complicated.” |
| **Patient- feelings on solving a puzzle in an app** | PT-PUZ | PT describes their feelings on solving a puzzle with their parent   - Interested | “If it was something you could almost select as an option to do.” |
| **Patient- availability of time to solve a puzzle game** | PT-PUZ-TIME | PT describes their availability of time they have to solve a puzzle game   - If it is fun, they would find the time | “I would say so, because most of your regular daily activities, you’re just being lazy--- Not everybody, but some people are being lazy and I would be able to find time in my day just to do something that will help me with something that’s important” |
| **Patient - length of puzzle game** | PT-PUZ-LENGTH | PT describes their thoughts on how long the puzzle game should take   - Depends on the type of game | “Two minutes?”  “No more than 15.”  “No more than five minutes.” |
| **Patient- types of games in app** | PT-GAME-TYPE | PT suggests types of games they would like to see in the app   - Tetris - Wordle - 20 questions | “I was thinking like maybe a Tetris, too, or I like just puzzles. Do like a small puzzle” |
| **Patient- feelings on parent collaboration in solving puzzle vs separately solving puzzle** | PT-COLLAB | PT describes their feelings on whether or not their parent should do the game with them (collaboration)   - Age dependent - Concern for parent having time | “Yes, I think that would be interesting to see. Yes.” |
| **Patient- type of app prompts** | PT-APP-PROMPT-TYPE | PT describes the type of prompts they would like to receive from the app   - Funny, ex. duolingo | “More of like “funny” prompt notifications versus something serious, just like, “Take your medicine”  [Patient]: Yes.” |
| **Patient- number of prompts from app** | PT-APP-PROMPT-NUM | PT describes the number of prompts from the app that would be burdensome   - Depends on medications - Less than 5 - Only when taking meds | “For the least, it’d just be when you need to take your meds.” |
| **Patient- daily single question on mood in app** | PT-APP-QUES | PT describes opinion on a single question prompt in the app   - I’m pretty comfortable - evening | “Maybe, but I guess I see a little problem with that. What if you were feeling a little crummy in the morning and you answered that, but then you had an amazing day?” |
| **Patient- rewards in app** | PT-REWARD | PT describes feelings on rewards in regards to medication adherence   - Very interested - Would feel nice to be rewarded | “A lot of it depends on when you started taking your meds, too, because if you have a chronic disease versus you just got diagnosed, a five-year-old might be like, “I don’t want to take these pills,” and might need a reward system to take it more willingly.” |
| **Patient- weekly survey in app** | PT-APP-SURVEY | PT describes feelings on answering surveys in the app   - Monthly would be better - Shorter survey with multiple choice - Only one short answer | “If it met all of the requirements that I wanted it to meet, like it reminding me, say, multiple times and reminding me at specific times when to take things, stuff like that, and it worked well, then I would definitely--- I'd rate it well.” |
| **Patient- AYA communication via app** | PT-APP-COMM | PT describes if they would like a leaderboard or chat feature with other AYAs in the app   - Chat feature - Talk to people in similar situations | “I feel like it should go on…but like everyone can still get their own points, but it’s just leaderboards that show how everyone is doing” |
| **Patient- Story 1 Advice** | PT-STRY-1 | PT gives their advice regarding story 1's scenario   - Tweak schedule - Wake him up - Put pill bottle on highly interacted with places | “If his schedule during the week allows it, just tweak the times. Say he doesn’t wake up at 9:00 a.m. on the weekends, if applicable to him and his case, try 11:00 a.m.” |
| **Patient- Story 2 Advice** | PT-STRY-2 | PT gives their advice regarding story 2's scenario   - Find a better sequence - Reach out to care team - Conversation regarding autonomy | “I would say pair her tacrolimus with something that makes her feel good. Usually, we just take our medicine with water or something, but maybe she has a favorite juice or a soda that she likes, like a Vernors, that kind of helps settle the stomach. I would say take it with your favorite beverage and that way you get a little bit of joy from it.” |
| **Patient- app feature suggestions** | PT-APP-FEAT | PT gives suggestion for app features   - Streaks - Accident forgiveness - Personalized feedback - Keeps track of multiple meds | “You could do like little animal emojis that are like your avatar.” |
| **Patient- app design suggestions** | PT-APP-DES | PT gives suggestion for app design   - Bright colors - Different modes - Customize | “Bright colors in the app. Something that’s not black and white. It can be more happy and more— Yes.” |
| **Patient- describes thoughts on notifications for medication log** | PT-LOG-NOTIF | Patient describes what they would do when prompted with a “new message” notification | “It’s definitely a way to make you open the app and it definitely works.” |
| **Patient- describes thoughts and actions on medication log** | PT-MED-LOG | Patient describes what they would do when logging medication and/or other thoughts about the log | “It all makes sense. The only thing that I look at and kind of wonder, “Oh, is that supposed to be a date, 7/15, for Monday?”” |
| **Patient- describes thoughts and actions on week log** | PT-WEEK-LOG | Patient describes what they would do when viewing weekly medication log and/or other thoughts about it | “In this case, what it looks like is you just click the week and then you are able to see the whole week, yes.” |
| **Patient- game view suggestions – notification** | PT-GAME-NOTIF | Patient provides suggestions on the notification associated with game view | “This notification, pretend this notification comes up on your phone. What would you do with it?  [Patient]: Click on it. “ |
| **Patient - game view suggestions - clue** | PT-CLUE | Patient provides suggestions on the clue associated with game view | “Then do you just click the box?” |
| **Patient - game view suggestions - how to play** | PT-HOW-PLAY | Patient provides suggestions on the how to play screen associated with game view | “I see the how to play, which it's pretty self explanatory. I assume you just click on it, and then it would show you how the game works or how you need to play the game, the rules, or something like that.” |
| **Patient - game view suggestions – puzzle screen** | PT-PUZZ-SCREEN | Patient provides suggestions on the puzzle screen associated with game view | “You would type in what you think the word is.” |
| **Caregiver- Reason AYA missed dose** | CG-REAS | Caregiver describes reasons for AYA missing dose.   - Completely forgot - Out with friends - Overwhelmed - Rebellious at this age - Complex situation - Avoidance | “The school days are busy in the morning, so it's very often and very easy to forget, “I have to take meds.”” |
| **Caregiver- Time of day AYA missed dose** | CG-TIME | Caregiver reports what time of day AYA is most likely to miss a dose and gives reasoning.   - Not awake yet - Fell asleep too early - During work hours | “He didn’t want to get up to take it. Then he worked, right. He had to take it in the morning. He’d be working. He’d drive a truck. He didn’t want to stop to take the medicine. Probably he was just skipping it.” |
| **Caregiver- Concerns with AYA missing dose** | CG-CONC | Caregiver describes their concerns regarding AYA missing a dose of their medication   - Panic, negative reaction - AYA not taking illness seriously - Poor short term memory | “For me, I think it's a little bit of distraction, but I think more if you don’t see him taking it or when he’s away, “Did you take it? Are you sure that you took it?” I think that's what’s more of a concern. Asking a number of times, making sure that he really took it or not? I think that's one, but other than that, yes. “ |
| **Caregiver- Communication with AYA regarding medication adherence** | CG-COMM | Caregiver describes their communication and interactions with AYA regarding medication adherence.   - Nagging - “Give them latitude” - Hovering - Let child give their input - Affects the relationship | “Yes, like, “As far as medications, let me know when you're ready to take it,” because sometimes she has a problem swallowing her medicine. That was a big issue, and I had to ask the doctor if she has to take it at this certain time, and if they say no, then I'm like, “Okay, well, you let me know when you're ready to take your medicine. Then you've got to let me know how you feel, and that way I won't be irritated or---”” |
| **Caregiver- Reaction to AYA missing dose** | CG-RXN | Caregiver describes how they react towards their AYA after they miss a dose.   - Yell at them - Be patient | “Sometimes I panic. Depending on what the medicine is for, I panic and then, even if it's late, if I remember, I'll get up and give it to her still.” |
| **Caregiver- Emotions surrounding caring for AYA with illness** | CG-EMOT-CARE | Caregiver describes their emotions surrounding caring for an AYA with an illness and helping with medication adherence.   - “Like juggling” - Overwhelming - Worn out | “It's my job.” |
| **Caregiver- Motivation to support AYA medication adherence** | CG-MOTIVE | Caregiver describes their motivation for supporting their AYA with medication adherence.   - “Everything we do is for our kids” - Keep kid healthy | “Yes, I’m just trying to keep her healthy.” |
| **Caregiver- Mental health concerns for AYA** | CG-MEN-HLTH-AYA | Caregiver describes their mental health concerns for their AYA when dealing with their illness and medications.   - Friends moving on - Not able to participate in school activities | “Sometimes I notice she's been through a lot and I know she gets tired, I just don't like, at the beginning she was willing to give up, she didn't want to go through the chemo, she didn't want to take the medicine. It was a fight and a struggle with her to get her started, but as we were getting through the progress and skipping the process, it was a struggle at first, but---” |
| **Caregiver- Knowledge on AYA treatment/medication** | CG-KNOWL-MED | Caregiver describes the type of knowledge on their AYA’s treatment and medication that would be helpful.   - Medication side effects - Consequences of missing a dose - Knowledge of where child’s numbers are at | “How does it impact the organs of your body because it has been a prolonged… It's not just like taking it for a year or six years, and you should continue to take it in the future, too. We would like to know a little bit more about… I know that it’ll be harder on kidneys, and everything, but I don’t know the detailed things like even though it impacts, what’s something better you can do so that the child doesn’t have to go through anymore complications.” |
| **Caregiver- AYA medication adherence improvement** | CG-AYA-IMPRO | Caregiver describes their opinion of their AYA’s improvement of medication adherence over time.   - “Balance between being a rebel and compliant” - Forming habits - Eventually want to do it on their own as get older | “She’s starting to help me” |
| **Caregiver- Strategy for medication adherence** | CG-STRAT | Caregiver describes the strategies they employ to help their AYA with medication adherence.   - Pill box - Phone alarm - Written out medication log | “I tried the pillbox. She does not want to deal with the pillbox.”  “It’s alarms.” |
| **Caregiver- Preference on involvement in AYA medication adherence** | CG-PREF-INVOLV | Caregiver describes their preference on whether or not they are involved in AYA’s medication adherence.   - Feel it is important | “I don’t worry about it that way. If I do it together, then I know she’s learning, so eventually she’ll be able to take over.” |
| **Caregiver- Feelings towards girlfriend/boyfriend assisting AYA in medication adherence** | CG-GF-BF-INVOLV | Caregiver describes their feeling towards their AYA’s girlfriend or boyfriends assisting with medication adherence   - Parent feeling taken for granted when AYA only listens to GF/BF advice - Nice to have a break | “It was nice, not going to lie, that little bit of a break, (laughter), even for a couple of hours at night.” |
| **Caregiver- Backup help with medication adherence** | CG-BACKUP | Caregiver explains whether having a backup person or system to help with AYA’s medication adherence is useful and who this backup person/system is.   - Other parent; Family members; Girlfriend or Boyfriend - Use of a different tone helpful - Peer support | “A girlfriend, or as was in my case, coming from the dad. Sometimes coming from the dad, especially for a guy, is another way of approaching it. They say it with a different tone or whatever. There’s always that new dynamic. It’s nice for that one mom or caregiver to have another backup. Just so you’re not always the one being the heavy.” |
| **Caregiver- Feelings regarding AYA medication compliance** | CG-AYA-COMP | Caregiver describes their feelings regarding AYA medication compliance   - “The kids are honest enough” - Trying their best | “They try their best. I don’t think he would purposely not take it, but I don’t know what the situation would be like if he was getting sick from it, if that would make a difference. It’s just a hassle right now.” |
| **Caregiver- Technology usage with medication adherence** | CG-TECH-ADH | Caregiver explains their opinion on use of technology with medication adherence.   - Alarms | “Texts. The best way to… I'm sure there are many apps out there but I just thought about texting. That was the easiest for me to communicate with her, but her phone was off so she wouldn't see that so I had to go to her room and remind her, and that's when the issues started. “ |
| **Caregiver- Technology for mainstream health management** | CG-TECH-HLTH-PERS | Caregiver describes the types of technology they personally use for mainstream health management.   - Track steps, blood pressure, food intake on apps - Tracking makes you more mindful | “I have it on my Samsung, there’s an app and it tracks my steps also. For a while I was tracking more than that with food intake and water and all that.” |
| **Caregiver- Technology for mainstream health management for AYA** | CG-TECH-HLTH-AYA | Caregiver describes the types of technology their AYA uses for mainstream health management.   - Pedometer - Step game with rewards | “He has been tracking his steps too. He’s like, “I’ve got 2500 steps today”. Just because they want to keep them moving” |
| **Caregiver- Opinion using app to track medication adherence** | CG-APP-FEEL | Caregiver explains their opinion on the use of an app to track medication adherence.   - Easy - Parent participation/communication - Betters habits | “I think it would be helpful. I’m not always going to be around to remind her, so I think, the older she gets, especially technology, the way it is now, kids rely a lot more on the apps and stuff, so I think that it would be really good for her to just know what she needs to take, at what time.” |
| **Caregiver- Knowledge of when AYA should skip dose** | CG-KNOW-SKIP | Caregiver explains how they know when their AYA should skip a dose of their medication.   - Clinic phone call | “I get a message from the doctor usually, or a call.” |
| **Caregiver- Feelings towards AYA needing to skip a dose** | CG-FEEL-SKIP | Caregiver describes their feelings surrounding their AYA needing to skip a dose.   - Grateful for physician knowledge - Get a break - Not burdened - Alter doses | “I don’t really feel one way or the other about it. It's just what it is.” |
| **Caregiver- Challenges when AYA needs to skip a dose** | CG-CHAL-SKIP | Caregiver explains any challenges or difficulties that may arise when their AYA needs to skip a dose.   - Only patient, not parent, receives the phone call - Lots of meds to balance | “It’s difficult for me to remember, because once you get out of the habit of doing it, then you forget. It takes a couple of days to get back into the routine. It just puts a glitch in your routine.” |
| **Caregiver- Opinion on new technology (eCap)** | CG-ECAP | Caregiver explains their opinion on usage of new technology, eCap.   - Difficult with multiple pills or multiple days - Would love to try something new | “Yes, I think if you’re pulling it out of just one bottle, that’s fine. It doesn’t seem to work if you’re doing multiple days.” |
| **Caregiver- feelings on solving a puzzle in app** | CG-PUZZ-FEEL | CG describes their feelings on solving a puzzle with the PT   - “fine with it” | “It would just depend on the day. It's hard to tell without knowing what it is or what it looks like.” |
| **Caregiver- feelings of burden from prompts/reminders** | CG-BURDEN | CG describes feelings of potential burden from prompts/reminders from app | “No, I wouldn’t see it as a burden, really. Maybe if there were constant reminders about the game, that would be a bit of a burden, but constant reminders about the medication would not be a burden. If anything, that would be a positive for me because that's something that I need.” |
| **Caregiver- availability of time to solve puzzle in app** | CG-PUZ-TIME | CG describes PT’s time for solving a puzzle   - Time is not an issue | “Yes, I don’t think time is the issue.” |
| **Caregiver- feelings on collaborating with AYA in app to solve puzzle** | CG-COLLAB | CG describes the level of teamwork desired in order to solve the puzzle   - Either way | “Either way, it's fine for us, I think. It should not be any big problem.” |
| **Caregiver- number or types of prompts from app** | CG-APP-PROMPT | CG describes frequency desired for puzzle solving   - Twice daily | “I’d say about twice daily.”  “Yes, twice, probably.” |
| **Caregiver- single question from app on mood** | CG-QUES | CG describes opinion on frequency of a single question prompt in app   - Once a day is fine | “That’d be fine, just once a day.” |
| **Caregiver- weekly survey in app** | CG-SURVEY | CG describes opinion on frequency of a survey in app   - Once a week is okay | “maybe once a week or twice a week, but every day could be too much.” |
| **Caregiver- concerns with app** | CG-APP-CONCERN | CG describes their concerns with the app   - If child is with friends - Teens not interested in doing it | “If she’s hanging out with her friends, she’s not going to want to stop and go, “Hang on, I’ve got to solve this puzzle in a minute and take my meds.” Do you know what I mean?” |
| **Caregiver- AYA medication adherence when inpatient** | CG-INPATIENT | CG describes medication adherence when AYA is inpatient and app usage   - Easy to adhere when inpatient - Nurses reminding | “When you’re with them, basically, you know and they know, too. It's all when they're away from you” |
| **Caregiver – Patient opinion on parent knowing if dose taken** | CG-KNOWS | CG discusses PT opinion on parent knowing through the app that a dose is taken   - Child would not mind | “My daughter wouldn't mind.” |
| **Caregiver- Story 1 advice** | CG-STRY-1 | Caregiver gives their advice in regards to story 1.   - Alarm - Sticky note on computer (visual) - In-game reminder | “He couldn’t log into the game unless he took his meds.” |
| **Caregiver- Story 2 advice** | CG-STRY-2 | Caregiver gives their advice in regards to story 2.   - Chart with rewards - Let patient weigh in - Work on it until it becomes a habit | “She can carry a nice, whatever she likes, kind of candy or anything. After taking the pill, it can substitute the taste of the pill and she can just have the candy taste, so even in her mind it would be refreshing too.” |
| **Caregiver- Suggestions for intervention app features** | CG-SUGG-FEAT | Caregiver describes suggestions for the intervention app features.   - Collect rewards (the kids are gamers) - streaks | “If we can go in the app and have the app send them a notification. Like, “Check and see if she took her meds today.” I think that would be something.” |
| **Caregiver- Participation in app intervention** | CG-APP-PAR | Caregiver explains if they would also participate in the app intervention, along with their AYA   - Willing to participate | “How do you feel about also being on that same application that the patient is?  Care Partner: It’s great.” |
| **Caregiver- daily medication log suggestions** | CG -DAY-LOG | Caregiver provides suggestions on the daily medication log | ““Thursday morning dose, evening dose…” What if they take night doses too?” |
| **Caregiver- weekly view suggestions** | CG-WEEK-LOG | Caregiver provides suggestions on the weekly view | “Now let’s say you want to go to “week view,” how would you go from there?  Care Partner: You would scroll over to your right. |
| **Caregiver- month view suggestions** | CG-MONTH-LOG | Caregiver provides suggestions on the month view | “If you press the month, what would you expect would happen?  Care Partner: It'll zoom out more, just like you’re peeling back. You'd see the whole month with two little things on each of them.” |
| **Caregiver- game questions/comments** | CG-APP-QUES | Caregiver asks questions and provides comments about the game | “I'm curious what the settings button leads to, what sort of things you can personalize or change to be helpful for you.” |
| **Caregiver- game view suggestions – notification** | CG-GAME-NOTIF | Caregiver provides suggestions about the notification associated with game view | “You would get this hangar notification on the phone, and after reading it, what would you want to do?  Care Partner: Tap on it to see.” |
| **Caregiver- game view suggestions - clue** | CG-CLUE | Caregiver provides suggestions about the clue associated with game view | ““Tap on the card to see your clue.” You tap on the card.” |
| **Caregiver- game view suggestions - how to play** | CG-HOW-PLAY | Caregiver provides suggestions about the how to play screen associated with game view | “Do you just tap “how to play”?  Interviewer: Once you tap “how to play,” you will see this screen.” |
| **Caregiver- game view suggestions – puzzle screen** | CG-PUZZ-SCREEN | Caregiver provides suggestions about the how to puzzle screen associated with game view | “There should be a “solve” button that you can fill in somehow.” |
| **Caregiver - child wanting autonomy in MA** | CG-AUTO | Caregiver describes their child wanting autonomy in medication adherence | “The doctors, they respected her wishes and what she wanted to take or what she didn't want to take. I think she liked when the doctors asked her.” |
