# Supplementary material for: Exploring Strategies for a Digital Tool to Support Medication Adherence Among Adolescents and Young Adults Undergoing Hematopoietic Stem Cell Transplant and Their Care Partners: Qualitative Formative Study
Source: JMIR Form Res. 2026 Feb 17;10:e82356. doi: 10.2196/82356 (PMC12957942; doi:10.2196/82356)
Supplement: Multimedia Appendix 4 [file formative_v10i1e82356_app4.docx]

**Multimedia Appendix IV: Patient and Care Partners Demographic Information – Individual and Dyadic Interviews**

| Gender | Number of Patients | Number of Care Partners | Total |
| --- | --- | --- | --- |
| Male | 3 | 1 | 4 |
| Female | 6 | 7 | 13 |
| Age | Number of Patients | Number of Care Partners | Total |
| <18 | 5 | 0 | 5 |
| 18-30 | 2 | 0 | 2 |
| 31-45 | 2 | 4 | 6 |
| 46-65 | 0 | 4 | 4 |
| Race | Number of Patients | Number of Care Partners | Total |
| Black or African American | 4 | 2 | 6 |
| white | 4 | 6 | 10 |
| Asian | 0 | 0 | 0 |
| Multiple Races | 1 | 0 | 1 |
| Ethnicity | Number of Patients | Number of Care Partners | Total |
| Hispanic or Latino | 0 | 0 | 0 |
| Non-Hispanic or Latino | 9 | 8 | 17 |
| Totals | 9 | 8 | 17 |
