# Supplementary figures and images for: Exploring Strategies for a Digital Tool to Support Medication Adherence Among Adolescents and Young Adults Undergoing Hematopoietic Stem Cell Transplant and Their Care Partners: Qualitative Formative Study
Source: JMIR Form Res. 2026 Feb 17;10:e82356. doi: 10.2196/82356 (PMC12957942; doi:10.2196/82356)

**
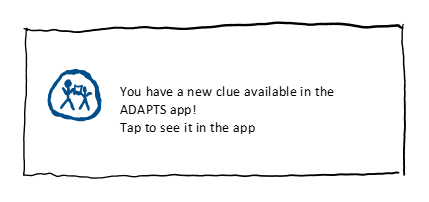

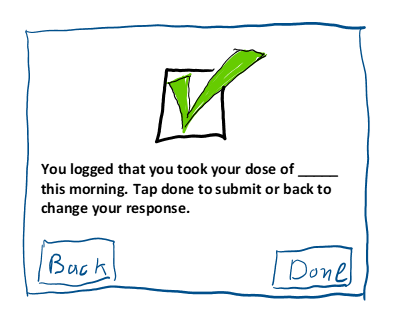

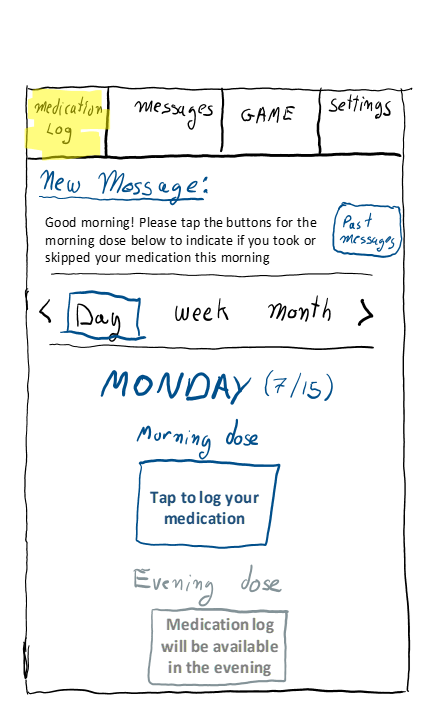

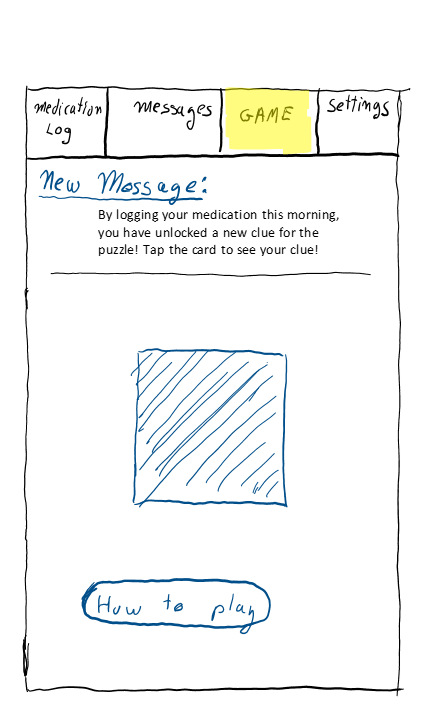

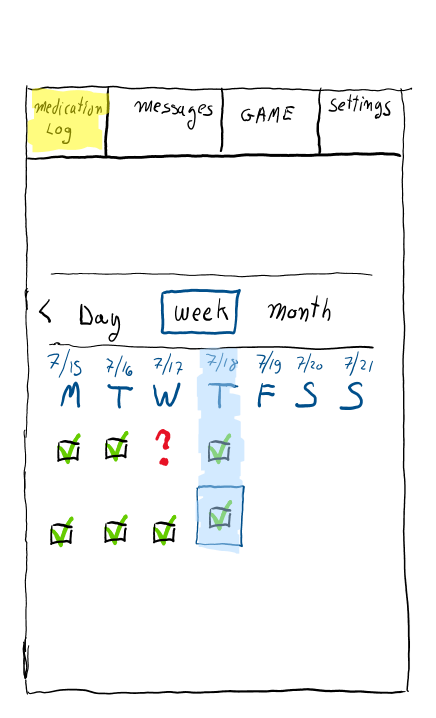

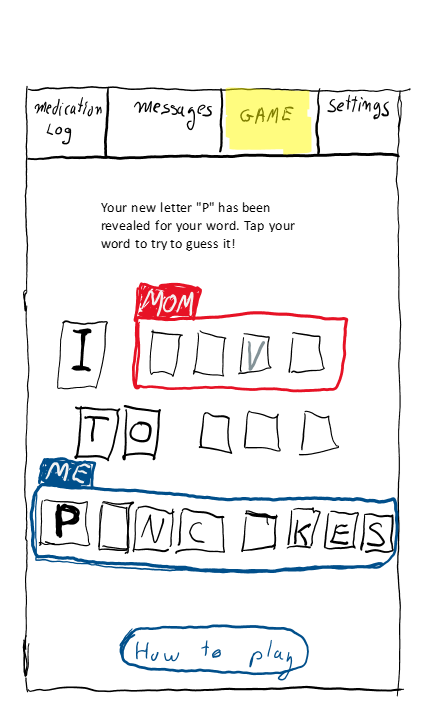
Multimedia Appendix V: Low-Fidelity, Paper-Based** **App** **Mock-Ups**

Supplement: Multimedia Appendix 5 [file formative_v10i1e82356_app5.docx]
